# Characterisation and elicitor properties of extracellular polymeric substances (EPS) obtained from cultures of *Sarocladium strictum* isolated from rye rhizosphere

**DOI:** 10.1007/s00203-025-04520-y

**Published:** 2025-10-21

**Authors:** Artur Nowak, Renata Tyśkiewicz, Iwona Komaniecka, Anna Pawlik, Grzegorz Janusz, Jolanta Jaroszuk-Ściseł

**Affiliations:** 1 Department of Industrial and Environmental Microbiology, Institute of Biological Sciences, Maria Curie-Skłodowska University, Akademicka 19, 20-033 Lublin, Poland; 2 Łukasiewicz Research Network–New Chemical Syntheses Institute, Analytical Laboratory, Tysiąclecia Państwa Polskiego Ave. 13A, 24-110 Puławy, Poland; 3 Department of Genetics and Microbiology, Institute of Biological Sciences, Maria Curie-Skłodowska University, Akademicka 19, 20-033 Lublin, Poland; 4 Department of Biochemistry and Biotechnology, Institute of Biological Sciences, Maria Curie-Skłodowska University, Akademicka 19, 20-033 Lublin, Poland

**Keywords:** Elicitors, Endophytic fungi, Extracellular polymeric substances (EPS), Plant resistance enzymes, Plant resistance induction, Culture optimisation

## Abstract

The rhizosphere is a habitat for a variety of microorganisms that can potentially be used for crop protection. Endophytic fungi are of particular interest because of their intimate interactions with plant roots. Nevertheless, a persistent challenge remains in the adaptation of microbial cultures to dynamic soil conditions, thus emphasising the importance of identifying strains capable of producing metabolites with elicitor activity. Extracellular polymeric substances (EPS) have been identified as promising candidates in this regard, given their ability to stimulate plant immune responses. The present study focused on optimising the production of. Extracellular polymeric substances (EPS) produced by *Sarocladium strictum* Th32Ag3 and evaluating its biological activity. The cultivation of the organism in sucrose-peptone medium resulted in a yield of 1.17 g/L of exopolysaccharide (EPS), consisting of 56% sugar cores, which were primarily composed of glucose, galactose, and mannose. The application of EPS to wheat seeds resulted in a substantial enhancement of defense-related enzyme activity, including phenylalanine lyase, tyrosine lyase, β-glucanase, and chitinase, without exerting any influence on oxidative stress enzymes such as catalase and peroxidase. Furthermore, EPS treatment resulted in a 30% increase in plant fresh weight. The elicitor effect of EPS was comparable to or even superior to that of chitosan, thus highlighting its potential as a sustainable biostimulant for strengthening crop resistance.

**Graphical abstract:**

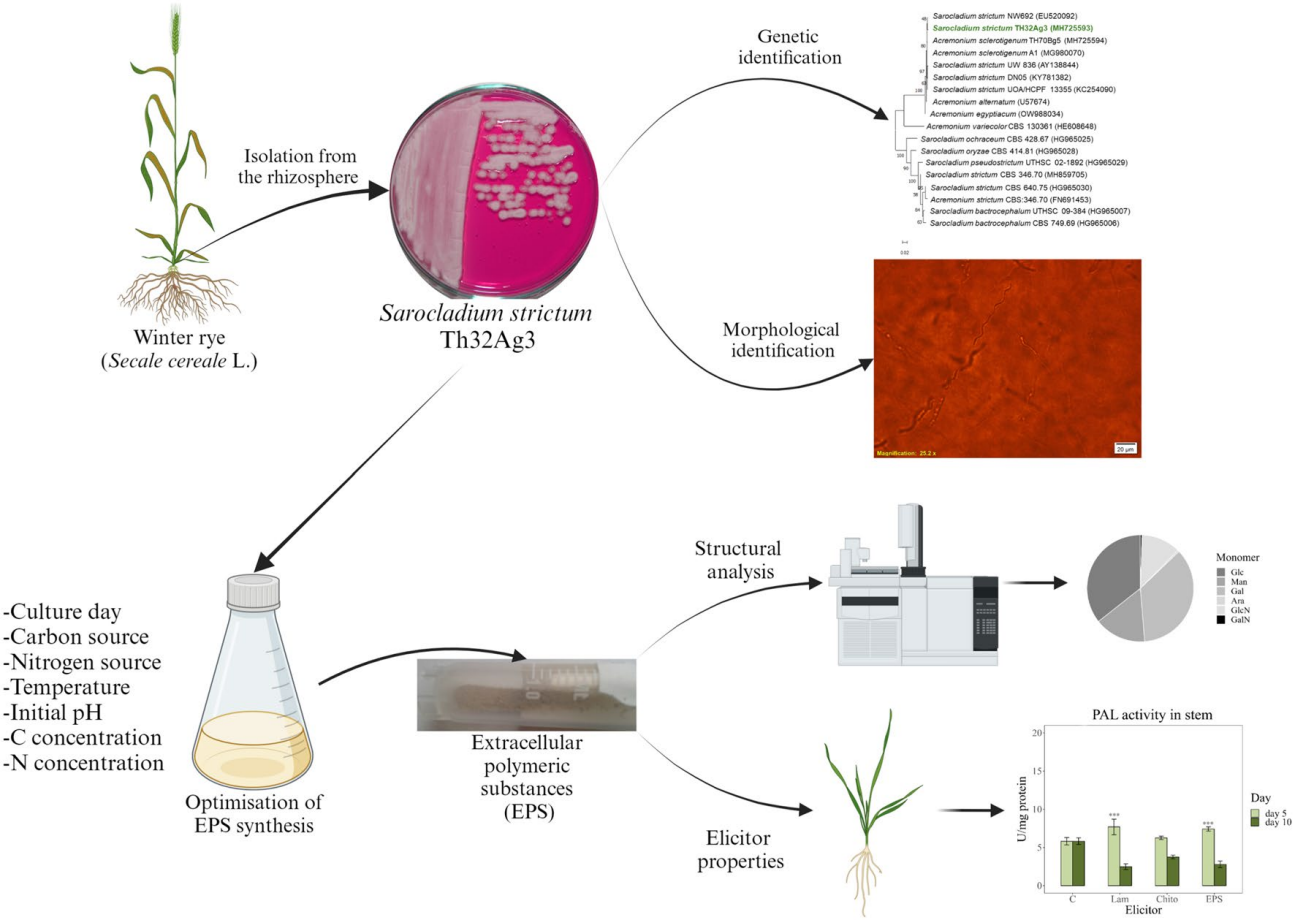

**Supplementary Information:**

The online version contains supplementary material available at 10.1007/s00203-025-04520-y.

## Introduction

Endophytic fungi reside in the root zones and plant tissues without harming the host and positively influence plant growth (Collinge et al. 2019; Sagita et al. 2021). Examples include *Trichoderma* spp., *Curvularia* spp., *Sarocladium* spp., and *Acremonium* spp., which interact with grasses, flax, tomatoes, maize, and wheat (Comby et al. 2016; Potshangbam et al. 2017; Tennant and Fermin 2018; El-Sayed et al. 2020; Khan et al. 2021; Tyśkiewicz et al. 2022). The mechanisms of beneficial effects on plants can be divided into two main groups: competition with phytopathogenic strains and the synthesis of bioactive compounds (Rojas et al. 2020). Endophytes are often categorised as Plant Growth-Promoting Fungi (PGPF) with applications in biostimulation and biocontrol (Khan and Umar 2021). They can interact directly with harmful microorganisms through processes such as competition, antibiosis, and mycoparasitism (Latz et al. 2018; Shamim et al. 2023). One of the main mechanisms influencing the interactions between endophytes and plants is their ability to synthesise a wide range of bioactive compounds (Usman et al. 2024). Bioactive compounds, including hormones such as indoleacetic acid (IAA) and gibberellins (GAs), which are crucial for growth and development from embryogenesis to senescence, protect plants against stress (Jenness et al. 2025). The capacity to synthesise phytohormones has been demonstrated in numerous fungal strains, including *Trichoderma* sp. (Jaroszuk-Ściseł et al. 2019), *Curvularia geniculata* (Priyadharsini and Muthukumar 2017) and *Paecilomyces formosus* (Khan et al. 2012). Siderophores, another significant compound group, bind iron ions (Poveda et al. 2021), with fungi synthesising hydroxamate and carboxylate-type siderophores such as coprogens, fusarinins, and ferrichromes (Chowdappa et al. 2020). Other mechanisms that classify endophytic fungi as potential PGPFs include their ability to solubilise phosphates, synthesise enzymes (1-aminocyclopropane-1-carboxylate (ACC) deaminase and chitinase) or directly stimulate plant immunity (Jaroszuk-Ściseł et al. 2019; Rojas et al. 2020; Adeleke et al. 2022).

The genus *Sarocladium* is well documented as a plant endophyte. Strains of this genus have been reported to inhibit the growth of phytopathogenic fungi, enhance plant growth and development, and boost pest resistance (Watts et al. 2023). A strain of *Sarocladium zeae*, a wheat endophyte that systemically colonises plants, has been demonstrated to confer protection against fusariosis (Kemp et al. 2020). *Sarocladium terricola* synthesises phytohormones from IAA and GA groups and siderophores (García-Latorre et al. 2023). In wheat grains under water-deficient conditions, two fungal endophytes, *Acremonium sclerotigenum* and *Sarocladium implicatum*, mitigate stress damage indicators and restrict the accumulation of stress-adaptation metabolites by altering 2-oxocarboxylic acid metabolism and regulating abscisic acid (ABA) levels (Llorens et al. 2019). A consortium of *Sarocladium* spp. fungi from *Agrostis stolonifera* stimulates the growth of *Festuca arundinacea* under lead-contaminated conditions (Soldi et al. 2020). Representatives of *Sarocladium strictum* have been shown to exhibit a number of mechanisms that increase their ability to interact with plants. A study conducted by Potshangbam et al. (2017) isolated strains of *S. strictum* from maize nodes and found them to possess the capacity for phosphate dissolution and the production of chitinase, protease, cellulase, amylase, and β-1,3-glucanase enzymes Additionally, these strains demonstrated the ability to synthesise siderophores and exhibited growth in a broad spectrum of pH, temperature, and salinity conditions. Another mechanism described to support plant growth is the ability to synthesise cis-13-octadecenoic acid, sebacic acid, pentamethoxy flavone, and n-hexadecanoic acid (palmitic acid), which have biocidal properties against insects (El-Sayed et al. 2020) It is evident that all of the aforementioned mechanisms have the capacity to exert a direct or indirect influence on plant life, thereby promoting growth and development (Zhang et al. 2023). Therefore, strains belonging to the genus Sarocladium have significant potential as biocontrol agents, further demonstrating their biostabilization and bioremediation capacities. An interesting feature of *Sarocladium* species is their lack of specificity towards the plant host. For example, a consortium containing Sarocladium spp. fungi isolated from the grass plant *Agrostis stolonifera* stimulated the growth of the grass plant *Festuca arundinacea* under Pb-contaminated conditions (Soldi et al. 2020). A similar relationship was observed between endophytic strains of *S. zeae*, which were isolated from maize and were capable of stimulating wheat resistance to *F. oxysporum* (Kemp et al. 2020). Furthermore, *Sarocladium* sp. representatives isolated from the soil under *Salicornia europaea* increased the salinity tolerance of *Lolium perenne* (Furtado et al. 2019). These findings suggest the high potential of Sarocladium isolates to protect other plant species against biotic and abiotic stress. However, there is a paucity of information in the extant literature regarding the ability of strains of the genus *Sarocladium* to synthesise extracellular polymeric substances (EPS).

EPS are macromolecules with a complex structure, which gives rise to a number of interesting properties (Siddharth et al. 2021; García et al. 2022; Nowak et al. 2025). The most significant component of EPS is its carbohydrate core, constituting 30–100% of the polymers' total weight (Andrew and Jayaraman 2020; Hamidi et al. 2022). It can be composed of a single monomer (homopolysaccharides) or different sugar residues forming defined subunits (heteropolysaccharides) linked by 1 → 2, 1 → 4, or 1 → 6 glycosidic linkages with α- or β-configurations. A linear structure can exhibit additional branching. In addition to sugars, EPS contains various substances, including proteins, phenolic compounds, amino sugars, uronic acids, and other compounds (Donot et al. 2012; Osińska-Jaroszuk et al. 2015; Siddharth et al. 2021). Some of the most significant properties of EPS are their capacity to aggregate soil particles, enhance the adhesion of microorganisms to root surfaces, function as carbon and nitrogen reservoirs, and stimulate plant immunity (Costa et al. 2018; Paul et al. 2024). The ability of fungal strains to synthesise EPS varies depending on several factors. A greater quantity of EPS was synthesised by pathogenic strains of *Fusarium culmorum* (1.1 g/L) than by non-pathogenic strains (0.2 g/L) (Jaroszuk‐Ściseł et al. 2020). Two *Plenodomus* sp. species demonstrated a correlation between virulence and EPS production, with the more virulent strain *Plenodomus lingam* exhibiting higher polymer production (0.43 g/L) than the less virulent *Plenodomus biglobosus* (0.29 g/L) (Nowak et al. 2023). One of the most interesting properties of fungal EPS is its ability to stimulate plant immunity (Frolova and Berestetskiy 2024). Commercially available polymers, such as laminarin from *Laminaria digitata*, are used for this purpose. Laminarin is a β-glucan that can stimulate pathogenesis-related (PR) proteins, defence enzyme activation, reactive oxygen species (ROS) burst or expression of genes responsible for plant immunity (Paris et al. 2019). Another polymer with similar properties is chitosan, which is a deacetylated chitin that stimulates immune pathways in plants (Chandrasekaran and Paramasivan 2024). Despite their high efficacy, it is important to identify new compounds with eliciting properties because of their price (laminarin) or preparation method (chemical deacetylation of chitin). Therefore, it is important to identify new sources of bioactive polymers (EPS).

This study aimed to ascertain the capacity of the *Sarocladium strictum* Th32Ag3 strain, which was obtained from the rhizosphere of a healthy rye (*Secale cereale* L.) plant, to synthesise exopolysaccharides (EPS). The study also aimed to optimise culture conditions to enhance the efficiency of EPS synthesis. These polymers have been shown to enhance a variety of plant resistance pathways in response to various biotic and abiotic stresses. Consequently, we investigated the direct interaction between EPS and wheat resistance pathways, encompassing both types of responses. The secondary objective was to confirm whether metabolites from an endophytic strain isolated from rye can influence the immune pathways of wheat. Furthermore, these results provide a promising basis for evaluating the potential of EPS induction to reduce plant infections by phytopathogens. The results presented here are believed to be the first report of the *S. strictum* strain's ability to synthesise EPS.

## Material and methods

### Sarocladium strictum Th32Ag3 strain identification

The strain *Sarocladium strictum* Th32Ag3 was isolated from the rhizosphere associated with the roots of winter rye (*Secale cereale* L.) grown near Lublin, Poland during the tillering period. This strain was morphologically identified. The strain was cultivated on Martin's medium with the following composition: Glucose 10 g/L (Chempur, Piekary Śląskie, Poland), peptone 5 g/L (BTL, Łódź, Poland), KH_2_PO_4_ 1 g/L (Chempur, Piekary Śląskie, Poland), MgSO_4_·7H_2_O 1 g/L (Chempur, Piekary Śląskie, Poland) and agar 15 g/L (Biomaxima, Lublin, Poland) supplemented with 1% streptomycin (POL-AURA, Zawroty, Poland) and 1% Rose Bengal (POL-AURA, Zawroty, Poland) after sterilization (Martin 1950; Jaroszuk-Ściseł et al. 2019).

The mycelia from 40 mL liquid cultures were used for DNA extraction according to the method of Borges et al. (1990). The purity and quantity of the DNA samples were evaluated using an ND-1000 spectrophotometre. PCRs was performed using Dream Taq Green PCR Master Mix. To confirm fungal identity, the ITS region in the nuclear ribosomal repeat unit was determined by direct sequencing of the PCR products amplified with ITS1-ITS4 primers, as described previously (White et al. 1990). Specific PCR products were purified using the Cleanup kit, followed by direct sequencing performed by Genomed S.A. (Warsaw, Poland). Sequencing data were analyzed with Lasergene v.8.0 software (DNASTAR, Inc., Madison, WI, USA). Database searches were performed using BLAST (Altschul et al. 1990). Multiple DNA sequence alignments were performed using the Clustal-W algorithm (Thompson et al. 1994). The Neighbour-Joining (NJ) algorithm was employed to construct a phylogenetic tree as implemented in MEGA v.11.0 software (Tamura et al. 2021). Sequences with the following accession numbers were used to construct the phylogenetic tree: EU520092, MH725594, MG980070, AY138844, KY781382, KC254090, U57674, OW988034, HE608648, HG965025, HG965028, HG965029, MH859705, HG965030, FN691453, HG965007 and HG965006. The topology of the tree was evaluated using bootstrap analysis of the sequence data based on 500 random resamplings. The GenBank accession number assigned to the nucleotide sequence determined in this study was MH725593.

### Screening tests for growth-promoting properties (PGP)

The tested isolate was cultivated on RB medium, which is composed of the following ingredients: Glucose: 10 g/L (Chempur, Piekary Śląskie, Poland); KH₂PO₄: 1 g/L (Chempur, Piekary Śląskie, Poland); MgSO₄ × 7H₂O: 0.5 g/L (POCH, Gliwice, Poland); KCl: 0.5 g/L (POCH, Gliwice, Poland); 0.5 g/L (NH₄)₂SO₄ (POCH, Gliwice, Poland); and 1 ml of a microelement solution (composed of 100.0 mg of Na₂B₄O₇ × 10H₂O (Sigma-Aldrich, Hamburg, Germany) and 10.mg CuSO₄ × 5H₂O (POCH, Gliwice, Poland), 50.0 mg FeSO₄ × 7H₂O (POCH, Gliwice, Poland), 10 mg MnSO₄ × 5H₂O (POCH, Gliwice, Poland) and 10 mg MgSO₄ × 7H₂O (POCH, Gliwice, Poland), 10 mg (NH₄)₆Mo₇O₂₄ × 4H₂O (Sigma-Aldrich, Hamburg, Germany) and 70.0 mg ZnSO₄ × 7H₂O (POCH, Gliwice, Poland) in 100 mL DW), supplemented with 3 mM tryptophan (sterilised by 0.2 µm filtration). The medium was inoculated with a spore suspension (5 × 10^5^) and incubated for 5 days at 20 °C and 120 rpm (Jaroszuk-Ściseł et al. 2019). Subsequently, the biomass was separated from the culture liquid. The Fe^3^⁺ complexing compound content (Woźniak et al. 2023), IAA concentration (using the Salkowski method) (Salkowski 1885), phenolic compound content (using the Folin-Ciocalteu method) (Singleton et al. 1999) and chitinase and β-glucanase activity (based on the released n-acetylglucosamine and glucose) were then determined (Rodriguez-Kabana et al. 1983; Hope and Burns 1987; Jaroszuk-Ściseł et al. 2019). At the same time, plate screening tests were performed to determine proteolytic activity on a milk agar (200 ml skimmed milk and 15 g/L agar) (Chandran et al. 2014), amylolytic activity using starch agar (10 g/L starch (POCH, Gliwice, Poland), 0.5 g/L KH₂PO₄ (Chempur, Piekary Śląskie, Poland), 0.5 g/L K₂HPO₄ (Chempur, Piekary Śląskie, Poland), 0.2 g/L MgSO₄ × 7H₂O (Chempur, Piekary Ślaskie, Poland), (NH₄)₂SO₄ × 0.2 g/L (POCH, Gliwice, Poland) and agar 15 g/L (Biomaxima, Lublin, Poland) (Khokhar et al. 2011)), and siderophores production on CAS agar (glucose 4 g/L (Chempur, Piekary Śląskie, Poland); KH₂PO₄ 3 g/L (Chempur, Piekary Śląskie, Poland); NaCl 0.5 g/L (POCH, Gliwice, Poland), 1 g/L NH₄Cl (POCH, Gliwice, Poland), 0.2 g/L f MgSO₄ × 7H₂O (Chempur, Piekary Śląskie, Poland) and 15 g/L agar (Biomaxima, Lublin, Poland) was dissolved in 860 ml of 0.1 M PIPES buffer (Merck, Darmstadt, Germany). The following solutions were used: 10% acidic casein hydrolysate (POCH, Gliwice, Poland) (30 ml); 0.01 M CaCl₂ (POCH, Gliwice, Poland) (10 ml); and a dark blue CAS-complex solution (100 ml), prepared by mixing 60.5 mg of Chromazurol S (CAS) (Fulka, Gothenburg, Sweden) in 50 mL of 10 mM HCl (POCH, Gliwice, Poland), 10 mL of 1 mM FeCl₃ × 6H₂O (POCH, Gliwice, Poland), and 40 mL of 72.9 mg of detergent (hexadecyltrimethylammonium bromide (HDTMA) (Sigma-Aldrich, Hamburg, Germany). All solutions were then mixed after sterilisation) (Schwyn and Neilands 1987). All plates were incubated for 72 h at 20 °C. The diameters of the colonies and zones obtained were determined, and the effectiveness was calculated according to the formula:

E = ØEz/ØFc

where E—effectiveness; ØEz—diameter of Echo zone; ØFc—diameter of fungal colony.

E < 1 – Zone smaller than the colony.

E = 1 – Zone the size of the colony.

E < 1 – Zone larger than the colony.

To determine the properties promoting plant growth (PGP), tests were performed to determine plant growth in the presence of the tested strain *S. strictum* Th32Ag3. Wheat seeds were surface-sterilised using 0.1% HgCl₂ and then placed in two-chamber Phytotoxkit sets (TIGRET, Warsaw, Poland). These sets contained 80 g of sterile soil with a moisture content of 60%. Ten wheat seeds were then laid out along the centre edge, and a spore suspension at a concentration of 1 × 10^4^ spores/g of soil was introduced. The control sample was treated with equivalent amounts of distilled water. The plants were then incubated for four days at 20–25 °C with a 12/12 h photoperiod. Subsequently, the length (mm) and dry weight (mg) of the stems and roots were determined.

### Optimisation of EPS synthesis

The optimisation of EPS synthesis was carried out using Czapek-Dox medium, with a starting composition comprising glucose (30 g/L) (Chempur, Piekary Śląskie, Poland), peptone (2.5 g/L) (BTL, Łódź, Poland), K_2_HPO_4_ (1 g/L) (Chempur, Piekary Śląskie, Poland), MgSO_4_ × 7H_2_O (0.5 g/L) (POCH, Gliwice, Poland), FeSO_4_ × 7H_2_O (0.01 g/L) (POCH, Gliwice, Poland), KCl (0.5 g/L) (POCH, Gliwice, Poland), and NaNO_3_ (3 g/L) (POCH, Gliwice, Poland). The initial pH was set at 7.0, and the temperature was maintained at 20 °C with a shaking speed of 120 rpm. A step-by-step optimisation process was conducted according to the following scheme (Fig. S1): 1. Length of cultivation: 2–11 days; 2. Carbon sources: sucrose (Chempur, Piekary Śląskie, Poland), glucose (Chempur, Piekary Śląskie, Poland), fructose (Sigma-Aldrich, Hamburg, Germany), mannose (Sigma-Aldrich, Hamburg, Germany); 3. nitrogen sources: peptone (BTL, Łódź, Poland), yeast extract (POCH, Gliwice, Poland), NH_4_NO_3_ (POCH, Gliwice, Poland), and (NH_4_)_2_SO_4_ (POCH, Gliwice, Poland); 3. Temperature (°C): 12; 20; 28; 4. Initial pH value: 4.5; 7.0; 9.5; 5. Carbon source concentrations (g/L): 3.75; 15; 30; 55; 187.5; 6. Nitrogen source concentrations (g/L): 1.2; 2.5; 7.5; 20; 60.

EPS was precipitated from the culture liquid using 96% ethanol (Linegal Chemicals, Blizne Łaszczyńskiego, Poland), in proportion of 1:3 (v/v), at 4 °C for 48 h. The resulting precipitate was separated from the supernatant by centrifugation (10,000 rpm, 4 °C, 15 min) and dried on an Eppendorf Concentrator Plus vacuum evaporator (three cycles of 5 h, 30 °C, 2,000 rpm, AQ mode, and aqueous solutions). The resulting EPS mass was expressed in g/L. The mycelia obtained were dried over three cycles (8 h, 85 °C), weighed, and expressed in g/L.

(Nowak et al. 2025).

Following the completion of the optimisation steps, a large-scale culture was conducted to obtain EPS for further analysis. The test isolate was cultured in optimised Czapek-Dox medium, yielding 10 L of culture liquid. The resulting liquid was concentrated five-fold and then precipitated with ethanol at a ratio of 1:3 (v/v). The resulting EPS was then subjected to centrifugation (10,000 rpm, 4 °C, 15 min) and subsequently lyophilised.

### Sugar component content and solubility of the resulting EPS

To determine the sugar content, the obtained EPS (1 mg/mL) was hydrolysed in 1 M sulphuric acid (VI) (POCH, Gliwice, Poland) at 100 °C for 8 h. The hydrolysate obtained was neutralised using 80 mg of BaCO_3_ (POCH, Gliwice, Poland).

The resulting EPS was suspended in water at 1 mg/mL and then shaken for 48 h at 20 °C at 120 rpm. After this time, the samples were centrifuged, and the soluble components in the resulting supernatant were determined: proteins by the Bradford method (1976), sugars by the Dubois method (1956), phenolic compounds by the Folin–Ciocalteau method (1999), uronic acids and amino sugars according to Madla et al. description (2005).

### Structure of the obtained EPS

#### Sugar components analyses by GC–MS

The EPS sample (2 mg) was hydrolysed in 2 M TFA (Merck, Darmstadt, Germany) for 4 h at 100 °C, dried, and N-acetylated using anhydrous methanol (POCH, Gliwice, Poland), pyridine (Sigma-Aldrich, Hamburg, Germany), and acetic anhydride (Sigma-Aldrich, Hamburg, Germany) (5:1:1; v/v/v) at room temperature for 18 h (Que et al. 2000). The reagents were removed under a nitrogen stream, and the sample was reduced with NaBD_4_ (Sigma-Aldrich, Hamburg, Germany) (room temperature, 18 h), acidified with glacial acetic acid (Sigma-Aldrich, Hamburg, Germany), and dried. The borates were removed by distillation of the sample with 5% acetic acid (POCH, Gliwice, Poland) in methanol (POCH, Gliwice, Poland) (3 times) and with methanol (POCH, Gliwice, Poland). The sample was acetylated with acetic anhydride (Sigma-Aldrich, Hamburg, Germany) and pyridine (Sigma-Aldrich, Hamburg, Germany) (1:1, v/v) at 85 °C for 30 min, and the reagents were removed by evaporation in a stream of nitrogen. The peracetylated (amino)alditol acetates were then recovered by extraction with a chloroform/water (1:1, v/v) (Sigma-Aldrich, Hamburg, Germany) mixture and found in the organic (lower) phase, which was consecutively dried using anhydrous sodium sulphate and nitrogen stream. For uronic acid analysis, 2 mg EPS sample was subjected to methanolysis (2 M HCl/methanol, 85 °C, 18 h) (POCH, Gliwice, Poland) and carboxyl reduced with NaBD_4_ (Sigma-Aldrich, Hamburg, Germany) (4 °C, 48 h), acidified with glacial acetic acid (Sigma-Aldrich, Hamburg, Germany), and dried. After removing the borates, the sample was hydrolysed in 2 M trifluoroacetic acid (Merck, Darmstadt, Germany) for 4 h at 100 °C, dried, and converted into alditol acetate, as described above. Methylation analysis of the EPS sample was performed according to the Ciukanu and Kerek procedure (1984). In this case, 7 mg EPS sample was dissolved in anhydrous DMSO (POCH, Gliwice, Poland), deprotonated with powdered NaOH (POCH, Gliwice, Poland), and methylated using methyl iodide. The methylation products were recovered from the methylation mixture using chloroform, hydrolysed (2 M trifluoroacetic acid (Merck, Darmstadt, Germany) for 4 h at 100 °C), and converted into (amino)alditol acetates, as described above.

All obtained sugar derivatives were analysed by gas chromatography coupled with mass spectrometry (GC–MS) using an Agilent Technologies instrument (7890A gas chromatograph and 5975C XL EI/CI mass spectrometer) equipped with an HP-5MS capillary column (30 m × 0.25 mm). Helium was used as the carrier gas at a flow rate of 1 ml/min. The temperature program involved a 5-min initial phase at 150 C, followed by a 5 °C per min ramping phase to reach 310 °C, where it was maintained for another 10 min.

#### Fourier-transform infrared spectroscopy (FTIR)

FTIR analysis was performed on lyophilised EPS samples using a Nicolet 8700A FTIR spectrometer. The FTIR spectra were measured in the frequency range of 4000–400 cm^−1^, with a resolution of 4.0 cm^−1^ and a scanning rate of 320 scans/s (Jaroszuk‐Ściseł et al. 2020).

#### Microscopic visualization

##### Fluorescence microscopy

Calcofluor (200 µL) (Sigma-Aldrich, Hamburg, Germany) was added to 5 mg of the test polymers and incubated for 25 min in the dark. The solution was then centrifuged for 10 min at room temperature and 10,000 rpm, after which the resulting sediment was transferred to a glass slide and observed using an Olympus BX53 Upright Microscope equipped with an Olympus XC30 camera with excitation at 365 nm and emission at 435 nm.

##### Scanning electron microscopy (SEM)

The isolate was grown on Martin's medium, from which discs of 5 mm diameter were cut, with excess agar removed with a scalpel. The samples were then fixed with a mixture of 2% glutaraldehyde (Sigma-Aldrich, Hamburg, Germany) and 2.5% paraformaldehyde (Sigma-Aldrich, Hamburg, Germany) in 150 mM phosphate-buffered saline (PBS) at pH 7.0 for a period of 24 h. Thereafter, the samples were rinsed three times with Cacodilate buffer (100 mM; pH 7.2–7.4) (Sigma-Aldrich, Hamburg, Germany) for 15 min. Thereafter, the samples were rinsed three times with Cacodilate buffer (100 mM; pH 7.2–7.4) (Sigma-Aldrich, Hamburg, Germany) for 15 min. Subsequently, the samples were fixed with 1% osmium oxide in cacodilate buffer (Sigma-Aldrich, Hamburg, Germany) for 1 h. The samples were then rinsed twice in Cacodilate buffer (Sigma-Aldrich, Hamburg, Germany) for 15 min. The samples obtained were then dehydrated in a water–ethanol series in increasing ethanol (POCH, Gliwice, Poland) concentrations (30, 50, 70, 80, 90, and 99.8%), with each concentration maintained for 1 h. The resulting samples were then dried at the critical point, sputtered with gold particles and analysed using a scanning microscope (Cesário et al. 2021; Mahmoud et al. 2023).

### Induction of plant resistance

Marker enzyme activity was determined in the stems and roots of winter wheat (*Triticum aestivum* L.) cv. Arkadia. Wheat seeds were sterilised with 0.1% HgCl_2_ (POCH, Gliwice, Poland) for 7 min and washed five times with sterile distilled water. The experimental setup was based on three biological replicates. Each biological replicate consisted of 50 wheat seeds that germinated and grew in the presence of the tested elicitors.

Culture stimulation was performed using the following variants: 1. Control – water; 2. Positive control – 0.05% aqueous suspension of laminarin (Lam) (Sigma-Aldrich, St. Louis, MO, USA) or chitosan (Ch) (Sigma-Aldrich, St. Louis, MO, USA); 3. Test sample – 0.05% aqueous suspension of EPS obtained.

Subsequently, the surface-sterilised wheat seeds were transferred to a hydroponic culture, where they germinated and grew in the presence of the aforementioned elicitors. The culture was conducted in a climate chamber at 25 °C with standard 12/12-h light and 80% humidity. Wheat seedlings were harvested after 5 and 10 d of growth. The stems were then separated from the roots, and their fresh weight was determined. Weights of 1 g were prepared from the resulting plant material for further analysis (Nowak et al. 2022).

### Enzyme activity of plant resistance pathways

Enzymes were extracted from plant tissues according to the method described by Garsia-Limones et al. (2002) with some modifications (Nowak et al. 2022). One gram of stem or root was frozen in liquid nitrogen and ground into fine powder over a period of 2 min. Then, 8 mL of 50 mM phosphate buffer pH 7.5 with 1 mM EDTA (Sigma-Aldrich, Steinhim, Germany), 1 mM PMSF (Sigma-Aldrich, Steinhim, Germany), and 1% PVPP (Sigma-Aldrich, Steinhim, Germany) was added and ground for 1 min. The resulting plant tissue suspensions were transferred to Falcon-type tubes and centrifuged for 15 min at 10,000 rpm at 4 °C. The extracts thus obtained were separated into portions, frozen in liquid nitrogen, and stored at − 80 °C for further analysis. The samples were stored on ice between stages. The protein content was determined using the Bradford method (1976).

#### Determination of phenylalanine lyase activity (PAL)

To 100 µL of the sample, 500 µL of borate buffer pH 8.8 and 400 µL of 50 mM L-phenylalanine (Sigma-Aldrich, Steinhim, Germany) were added. After thorough mixing of the samples, the absorbance at 290 nm (T_0_) was measured, and the samples were incubated for 1 h at 37 °C. After this time, the absorbance at 290 nm (T_1_) was measured again. Subsequently, the difference between T_1_ and T_0_ was determined, and the amount of trans-cinnamic acid produced was determined from the calibration curve (Khan et al. 2003; Hanaka et al. 2022). PAL activity was determined as the amount of trans-cinnamic acid produced converted to 1 mg protein (U/mg protein).

#### Determination of tyrosine lyase activity (TAL)

To 100 µL of the sample, 500 µL of borate buffer pH 7.2 and 400 µL of 10 mM L- tyrosine (Sigma-Aldrich, Steinhim, Germany) were added. After thorough mixing of the samples, the absorbance at 310 nm (T_0_) was measured, and the samples were incubated for 1 h at 37 °C. After this time, the absorbance at 310 nm (T_1_) was measured again. Subsequently, the difference between T_1_ and T_0_ was determined, and the amount of p-coumaric acid produced was determined from the calibration curve (Khan et al. 2003; Hanaka et al. 2022). TAL activity was determined as the amount of p-coumaric acid produced converted to 1 mg protein (U/mg protein).

#### Determination of ascorbate peroxidase activity (APX)

To 100 µL of the sample, 400 µL of 50 mM phosphate buffer (pH7.0) and 250 µL of 1 mM ascorbic acid (Sigma-Aldrich, Steinhim, Germany) were added. The reaction was initiated by adding 250 µL of 20 mM H_2_O_2_ (POCH, Gliwice, Poland), and the absorbance was immediately measured at 290 nm for 1 min. APX activity was calculated from ε = 2.8 mM-1 cm-1and converted to 1 mg protein (U/mg protein) (García-Limones et al. 2002; Tyśkiewicz et al. 2024).

#### Determination of guaiacol peroxidase activity (GPX)

To 50 µL of the sample, 550 µL of 100 mM phosphate buffer (pH. 6.5) and 250 µL of 60 mM guaiacol (Sigma-Aldrich, St. Louis, MO, USA) were added. The reaction was initiated by adding 200 µL 0.25% H_2_O_2_ (POCH, Gliwice, Poland), and the absorbance was immediately measured at 470 nm for 1 min. GPX activity was calculated from ε = 26.6 mM-1 cm-1 and converted to 1 mg protein (U/mg protein) (García-Limones et al. 2002; Tyśkiewicz et al. 2024).

#### Determination of catalase activity (CAT)

To 100 µL of sample, 450 µL of 50 mM phosphate buffer (pH. 7.0). The reaction was started by adding 500 µL of 80 mM H_2_O_2_ (POCH, Gliwice, Poland) and the absorbance was immediately measured at 240 nm for 1 min. CAT activity was calculated from ε = 36 M-1 cm-1 and converted to 1 mg protein (U/mg protein) (García-Limones et al. 2002; Tyśkiewicz et al. 2024).

#### Determination of β-glucanase activity (β-GLUC)

To 100 µL of the sample (P_b_), 100 µL of 0.5% laminarin (Sigma-Aldrich, St. Louis, MO, USA) in 100 mM acetic buffer (pH 5.6) was added and incubated at 37 °C for 1 h with gentle shaking. Then, 100 µL of the reaction mixture was transferred to 400 µL of water and the content of released reducing sugars was determined using the DNS method (Miller 1959). In parallel with the test samples, a background sample (P_t_) (a plant extract sample heat-inactivated at 80 °C for 10 min) and a control sample (P_k_) were prepared under the same conditions. The amount of glucose released was calculated using the following formula:

$$ \mu {\text{molGlc}}\, = \,\left( {\left( {{\text{P}}_{{\text{b}}} - {\text{P}}_{{\text{k}}} } \right) - \left( {{\text{P}}_{{\text{t}}} - {\text{P}}_{{\text{k}}} } \right)} \right)/{\text{C}} $$

Where: P_b_—test sample, P_t_—background sample, P_k_—control, C—coefficient from the calibration curve (0.04708).

β-glucanase activity was converted per 1 mg protein (U/mg protein) (Khan and Umar 2021; Nowak et al. 2022).

#### Determination of chitinase activity (CHIT)

To 100 µL of the sample (P_b_), 100 µL of 0.5% colloidal chitin (Sigma-Aldrich, St. Louis, MO, USA) in 100 mM acetic buffer (pH 5.6) was added and incubated at 37 °C for 1 h with gentle shaking. Then, 100 µL of the reaction mixture was transferred to 400 µL of water and the content of released reducing sugars was determined using the DNS method (Miller 1959). In parallel with the test samples, a background sample (P_t_) (a plant extract sample heat-inactivated at 80 °C for 10 min) and a control sample (P_k_) were prepared under the same conditions. The amount of N-Acetyl-D-glucosamine released was calculated using the following formula:

$$ \mu {\text{molGlcNAc}}\, = \,\left( {\left( {{\text{P}}_{{\text{b}}} - {\text{P}}_{{\text{k}}} } \right) - \left( {{\text{P}}_{{\text{t}}} - {\text{P}}_{{\text{k}}} } \right)} \right)/{\text{C}} $$

Where: P_b_—test sample, P_t_—background sample, P_k_—control, C—coefficient from the calibration curve (0.00988).

Chitinase activity was converted per 1 mg protein (U/mg protein) (Khan and Umar 2021; Nowak et al. 2022).

### Statistical analysis

All the samples were separated into three independent replicates, which were then assayed in three independent assays, yielding a replicate count of n = 9. Data are presented as mean values with standard deviation (SD). The results were subjected to analysis of variance (ANOVA) followed by Tukey's post hoc test for multiple comparisons at p < 0.05. Enzyme activity was compared using the Dunnett's test, with the level of significance expressed as: ***—0.001; **—0.01; *—0.05;—0.1. The data were also analysed using PCA (Principal Component Analysis). All tests were performed using the open-source software RStudio for Windows version 2024.09.0 + 375 (Posit, PBC, GNU Affero General Public Licence v3).

## Results

### Morphological characteristics and phylogenetic position of Sarocladium strictum Th32Ag3 isolate

**Fig. 1:**
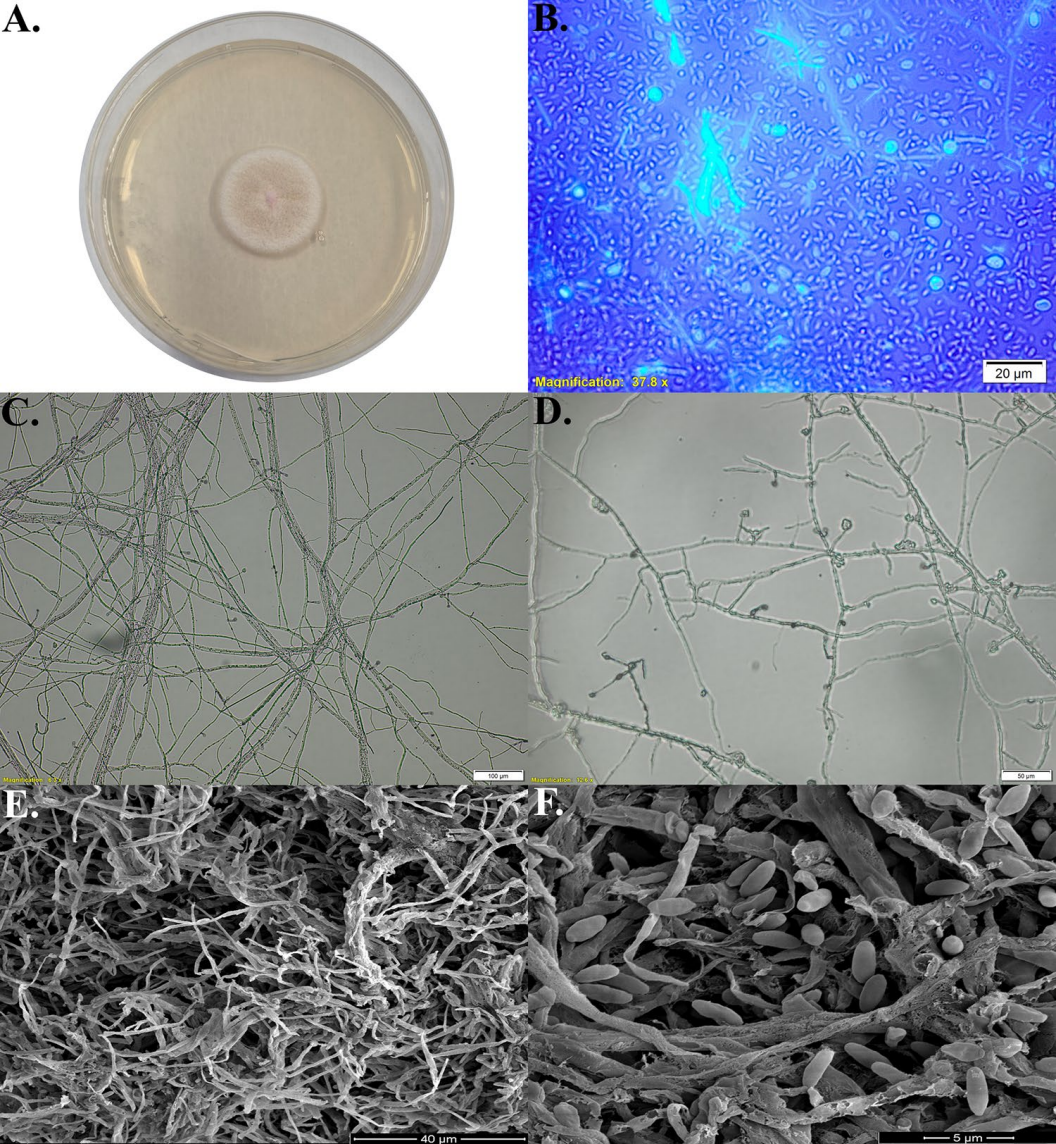
Macro/microscopic imaging of the strain *Sarocladium strictum* Th32Ag3: **A** Growth of *S. strictum* on PDA medium **B** Ellipsoidal Conidia stained with Calcolfuor-White, **C** Fungal hyphae visible under a light microscope, **D** Spore clusters on fungal hyphae, **E** Fungal hyphae visible under an SEM microscope, **F** Fungal hyphae with spores visible under an SEM microscope

The isolate exhibited characteristic white-cream growth on PDA medium, with a slow growth rate observed after 7 days of incubation (Fig. 1A). Spores derived from the culture were stained with calcofluor and displayed fluorescence under UV illumination, indicating the presence of chitin in the spore walls (Fig. 1B). The resulting image revealed oval spores, which is a characteristic feature of the genus *Sarocladium*. Light microscopy revealed both individual and intertwined hyphae forming densely packed clusters. The hyphae exhibited varying degrees of branching (Fig. 1C). Typical conidiophores terminating in phialides were also observed (Fig. 1D). SEM analysis confirmed these light microscopy findings, revealing septate, branched hyphae, and thick, twisted hyphal structures (Fig. 1E). The presence of oval spores at the tips of the conidiophores, which is characteristic of this species, was also confirmed (Fig. 1F).

**Fig. 2:**
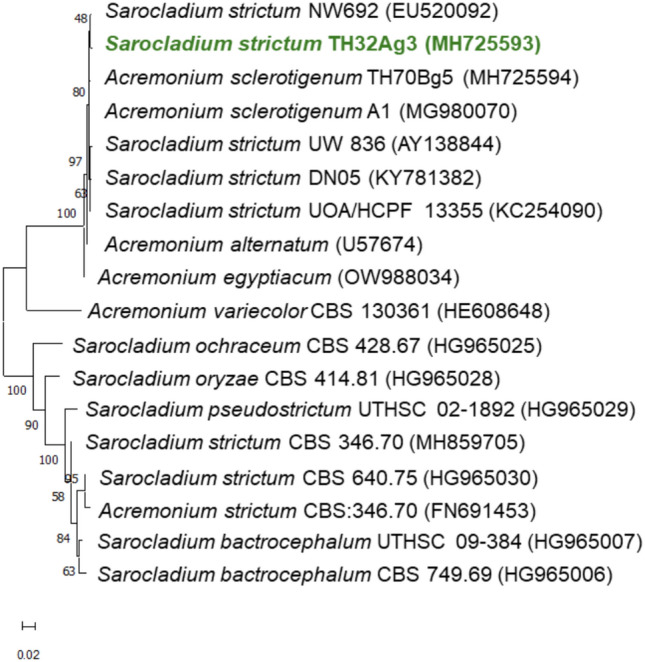
The evolutionary relationships of *Sarocladium*/*Acremonium*. The evolutionary history was inferred using the Neighbor-Joining method (Saitou and Nei 1987). The optimal tree is shown. The percentage of replicate trees in which the associated taxa clustered together in the bootstrap test are shown next to the branches (Felsenstein 1985). The tree is drawn to scale, with branch lengths in the same units as those of the evolutionary distances used to infer the phylogenetic tree. The evolutionary distances were computed using the Jukes-Cantor method (Jueks and Cantor 1969) and are in the units of the number of base substitutions per site. This analysis involved 18 nucleotide sequences. All ambiguous positions were removed for each sequence pair (pairwise deletion option). There were a total of 768 positions in the final dataset. Evolutionary analyses were conducted in MEGA11 (Tamura et al. 2021)

A product of 570 base pairs in length was obtained using PCR with the ITS1-ITS4 primers. Subsequently, the resulting reaction product was sequenced. The complete sequence of the product indicated 99% identity to the *Sarocladium strictum* ITS sequences. The GenBank accession number assigned to the nucleotide sequence determined in this study was MH725593. A phylogenetic tree was constructed for Th32Ag3 and other strains described in the databases using the NJ (Neighbor Joining) method (Fig. 2). The fungus clustered with other closely related *Sarocladium* and *Acremonium* species, forming a well-defined cluster that was further supported by a bootstrap value of 97%. In 2015 Giraldo et al. (2015) stated that *Acremonium* was reviewed on the basis of a DNA phylogenetic study and several species were subsequently transferred to *Sarocladium*. Even if both genera are morphologically similar though members of the order Hypocreales are phylogenetically distant, but still hardly to easily distinguish.

### Properties of the S. strictum Th32Ag3 strain

The tested strain exhibited the ability to synthesise several plant growth-promoting (PGP) compounds. In liquid cultures, the concentrations of indole-3-acetic acid (IAA), Fe-chelating compounds, and phenolic compounds reached 5.2 µg/mL, 103.7 µg/mL, and 206.7 µg/mL, respectively. Although the isolate did not display chitinolytic activity, it demonstrated considerable β-glucanase activity, quantified at 137.9 µg/mL. Plate screening revealed that the highest efficiency was observed for proteolysis, with an E value of 1.3 (Table 1A). In vivo assays with wheat seedlings inoculated with the isolated spores confirmed its growth-promoting effect. Compared to the control, inoculated plants exhibited an increase of approximately 40% in stem length and 42% in root length. Similarly, biomass accumulation was significantly enhanced, as indicated by a 34% increase in dry stem matter and a 62% increase in dry root matter (Table 1B).

**Table 1:** The plant growth-promoting properties of the *S. strictum* Th32Ag3 strain. Ability to synthesise plant growth-promoting compounds (indole-3-acetic acid (IAA), Fe-chelating compounds and phenolic compounds), chitinase and β-glucanase activity, and lytic activity (proteolytic and amylolytic), and ability to synthesise siderophores. B. Effect on the length and dry weight of wheat stems and roots

	A. Growth-promoting properties of the *S. strictum* Th32Ag3 strain							
Compoud	IAA	Fe-Chelating Compounds	Phenolic Compounds	Chitinolytic activity	β-glucanase activity	Proteolytic activity	Amylolytic activity	Siderophore production
Concentration	**5.2 ± 0.8 µg/mL**	**103.7 ± 4.5 µg/mL**	**206.7 ± 12.6 µg/mL**	**0**	**137.9 ± 11.4 µg/mL**	**1.3 ± 0.1** **E**	**0.7 ± 0.1** **E**	**0.3 ± 0.1** **E**

	B. The effect of the *S. strictum* Th32Ag3 strain on wheat seedling growth				
Plant characteristics		Stem length (mm)	Root length (mm)	Stem weight (mg)	Root weight (mg)
Water control		89.8 ± 1.3	71.8 ± 15.6	151.1 ± 3.6	66 ± 2.7
*S. strictum* Th32Ag3 inoculation		**112.2 ± 10.6**	**91.5 ± 3.8**	**196.3 ± 7.0**	**103.4 ± 3.4**

### Optimisation of EPS synthesis

The *S. strictum* strain demonstrated the capacity to synthesise EPS in Czapek-Dox medium. Following optimisation, an approximate 20% increase in the quantity of EPS obtained from the culture was observed, from 0.83 g/L to 1.17 g/L. The third day of the incubation period was identified as the optimal time point for EPS synthesis. The type of carbon source (sucrose) and concentration of the nitrogen source (7.5 g/L) exerted the greatest influence on the amount of EPS obtained (Table 2). However, there was no correlation between EPS concentration and dry mycelium mass. The highest biomass was observed after 5–11 days of culture growth, with an average level of 12 g/L. Further optimisation stages also indicated that alternative C and N sources are optimal for EPS synthesis and biomass yield. The highest biomass was obtained using fructose (11.33 g/L) and yeast extract (10.59 g/L). It is also worth noting that reducing the nitrogen source concentration to 1.2 g/L, which was associated with a significant increase in biomass to 21.24 g/L (Table S1).

**Table 2:** Results of step-by-step optimisation of *S. strictum* strain culture on the EPS yield (g/L)

	Optimised parameter																	
EPS yield g/L	**Length of cultivation**																	
	2	3	4			5		6		7	8			9		10		11
	0.38 ± 0.07 B	**0.83 ± 0.04** **a**	0.82 ± 0.1 a			0.23 ± 0.15 b		0 c		0 c	0 c			0 c		0 c		0 c
	**Carbon source**																	
	Sucrose				Glucose					Fructose					Mannose			
	**0.97 ± 0.02** **a**				0.88 ± 0.08 a					0.79 ± 0.12 a					0.53 ± 0.07 b			
	**Nitrogen source**																	
	Peptone				Yeast extract					NH_4_NO_3_					(NH_4_)_2_SO_4_			
	**1.01 ± 0.11** **a**				0.81 ± 0.06 b					0.57 ± 0.04 c					0.86 ± 0.06 ab			
	**Temperature (°C)**																	
	12						20						28					
	0.5 ± 0.01 b						**1.09 ± 0.02** **a**						0.6 ± 0.12 b					
	**Initial pH value**																	
	4.5						7.0						9.5					
	0.82 ± 0.02 b						**1.04 ± 0.08** **a**						0 c					
	**Carbon source concentrations (g/L)**																	
	3.75			15					30			55					187.5	
	0.27 ± 0.01 bc			0.36 ± 0.06 bc					**1.03 ± 0.03** **a**			0.19 ± 0.01 cd					0.14 ± 0.04 d	
	**Nitrogen source concentrations (g/L)**																	
	1.2			2.5					7.5			20					60	
	0.1 ± 0.01 d			0.95 ± 0.09 b					**1.17 ± 0.04** **a**			0.62 ± 0.06 c					0.21 ± 0.04 d	

Statistical analysis was performed by Anova test using Tukey post hoc p < 0.05

### Structure of the obtained EPS

**Fig. 3:**
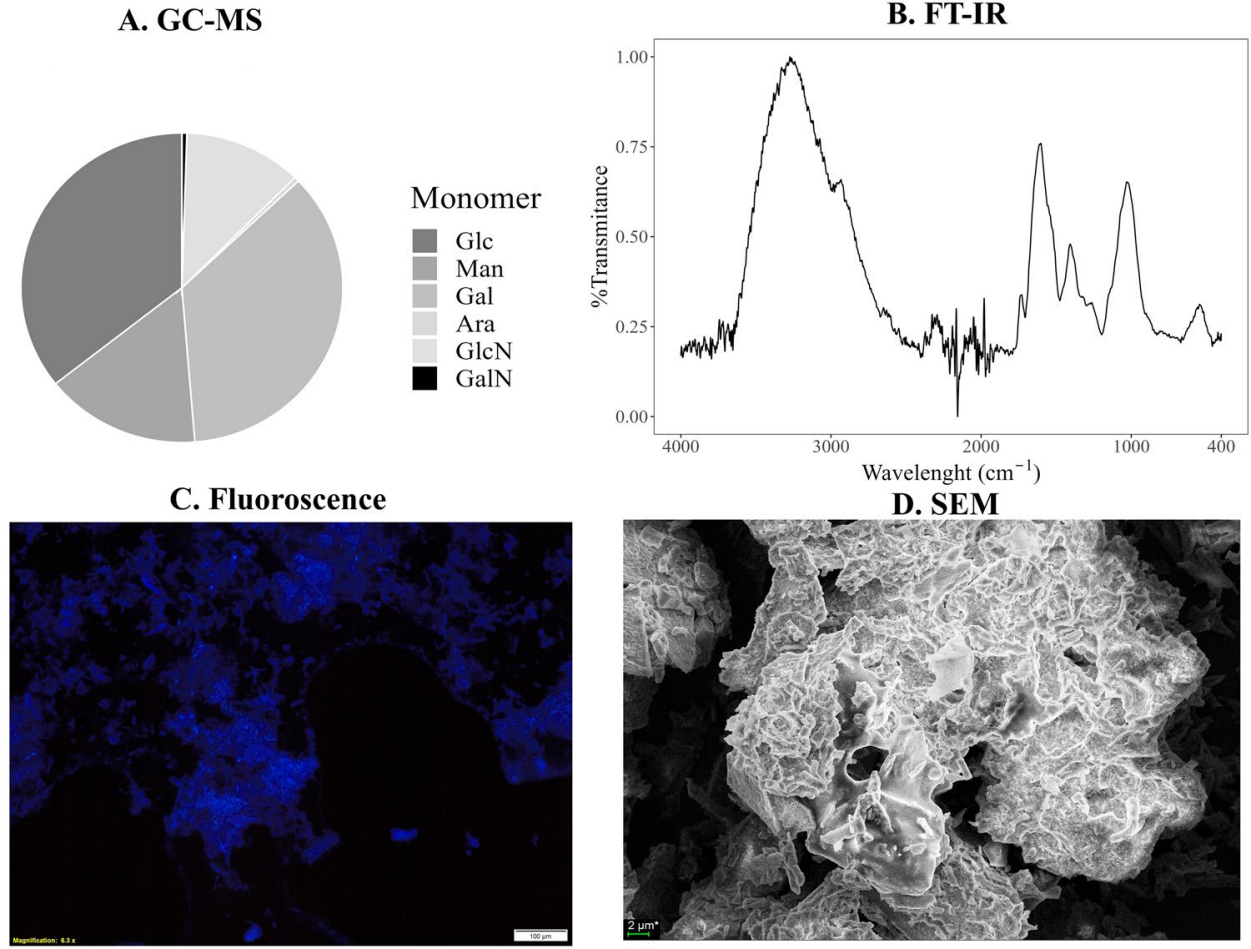
Structural characterization of the obtained EPS. **A** Composition of sugar monomers determined by GC–MS (*Glc* glucose, *Man* mannose, *Gal* galactose, *Ara* arabinose, *GlcN* glucosamine, *GalN* galactosamine), showing glucose and mannose as the dominant components. **B** FTIR spectrum of the obtained EPS in the 400–4000 cm⁻^1^ range, confirming the presence of characteristic polysaccharide functional groups. **C** Visualization of EPS obtained with calcofluor. **D** Surface morphology of EPS observed by SEM

The sugar core of the EPS represented 56.1% of the total weight of the polymers (Table 3B). The obtained polymers were incompletely water-soluble, with the following soluble components: sugars: 347.3 µg/mg, proteins: 3.4 µg/mg, phenolic compounds: 5.81 µg/mg, amino sugars: 3.58 µg/mg and uronic acids: 44.55 µg/mg (Table 3A). The sugar core of the studied EPS consisted mostly of galactose (36.6%), glucose (36%), mannose (15.8%), and trace amounts of arabinose (< 0.5%). Of interest is the presence of 12% glucosamine, which forms the sugar part, and traces of galactosamine (< 0.5%) (Fig. 2A). GC–MS analysis of the sample subjected to carboxyl reduction before acid hydrolysis and the conversion of liberated monosaccharides into alditol acetates revealed that glucuronic acid constituted 44% of the glucose, whereas galacturonic and mannouronic acids accounted for approximately 17% of the galactose and mannose pools. Methylation analysis revealed the presence of terminal hexose, pentose, and hexosamine residues, as well as 3-linked, 4-linked, and 6-linked hexose residues (Table 4). The spectroscopic and microscopic data were consistent with the GC–MS findings. The broad FTIR absorption at 3400–3200 cm⁻^1^ and the band at 1025 cm⁻^1^ correspond to O–H and C–O–C vibrations typical of glycosidic bonds, which aligns with the high proportion of glucose, galactose, and mannose detected by GC–MS (Shurvell 2006). The band at 1610 cm⁻^1^, usually associated with amide I, may also reflect the presence of amino sugars such as glucosamine and galactosamine revealed by GC–MS, further supporting the hybrid polysaccharide nature of EPS (Fig. 3B). Calcofluor staining confirmed the abundance of β-linked polysaccharides, consistent with the methylation analysis showing terminal and 3-, 4-, and 6-linked hexose residues. Moreover, the aggregation and irregular surface morphology observed under SEM can be related to the high uronic acid content detected by GC–MS, as the carboxyl groups promote intermolecular interactions and cross-linking (Fig. 3C, 3).

**Table 3:** Biochemical composition of the EPS obtained. A. Content of water-soluble components. B. Amount of the sugar core in the obtained EPS

Biochemical composition of EPS						
	A. Water-soluble components of EPS					B. Total sugar content in EPS
	Sugar	Protein	Phenols	Amino sugars	Uronic acids	
Concentration (µg/mg)	347.33 ± 27.86	3.4 ± 0.42	5.81 ± 1.56	3.58 ± 0.95	44.55 ± 7.54	561.59 ± 23.6

**Table 4:** Linkage analysis of EPS. EPS samples were methylated, hydrolyzed, reduced and acetylated. The obtained partly methylated alditol acetates were identified by GC–MS, based on their characteristic mass spectra and retention times

Retention time [min.]	Component	Component conten [%]
10.922	t-Pen	17
11.943	t-Hex I	42
12.424	t-Hex III	1
13.894	→ 3)-Hex	13
14.192	→ 4)-Hex	9
14.553	→ 6)-Hex	16
18.150	t-HexN	19

### Evaluation of plant resistance inducing properties

**Fig. 4:**
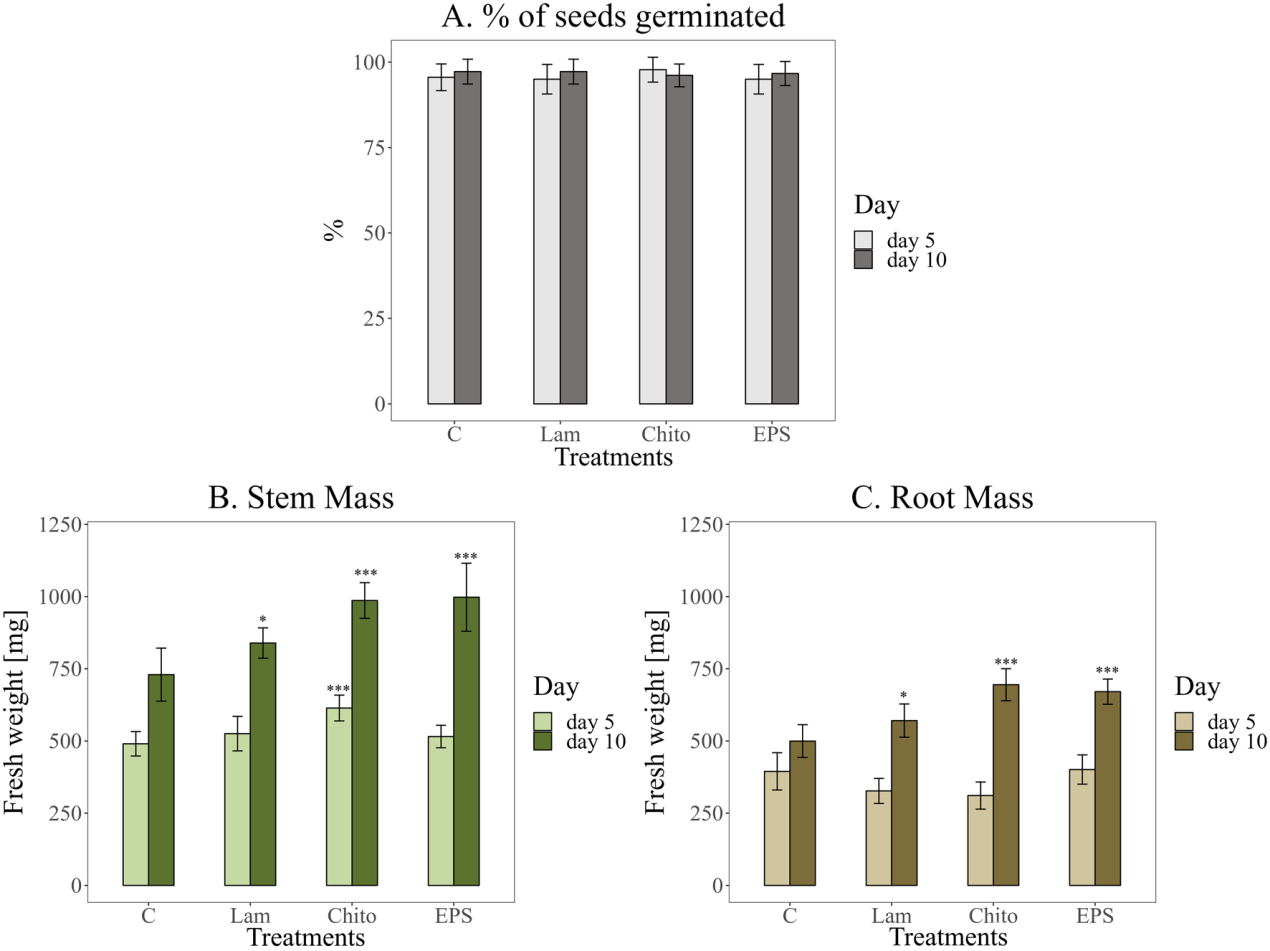
Effect of the addition of elicitors (C—water control, Lam—laminarin, Chito—chitosan, EPS—EPS from Th32Ag3 strain) on various wheat growth parameters. **A** % of seeds germinated, **B** Fresh weight of stems, C. Fresh weight of roots, after 5 and 10 days of plant growth. Significance against control determined by Dunnett's test, where p value is: *** 0.001; ** 0.01; * 0.05;**.** 0.1. The most pronounced increase in wheat fresh weight was observed after 10 days of growth in response to chitosan and EPS treatment

The applied elicitors did not negatively affect wheat seed germination. The percentage of germinated seeds was similar after 5 and 10 days, according to the producer's claims of ~ 95%. However, in the case of inoculation of wheat seeds with chitosan (after 5 and 10 days) and EPS (after 10 days), a significant increase in the fresh weight of the stems was observed. A similar relationship was observed for wheat roots after 10 d of plant growth (Fig. 4).

**Fig. 5:**
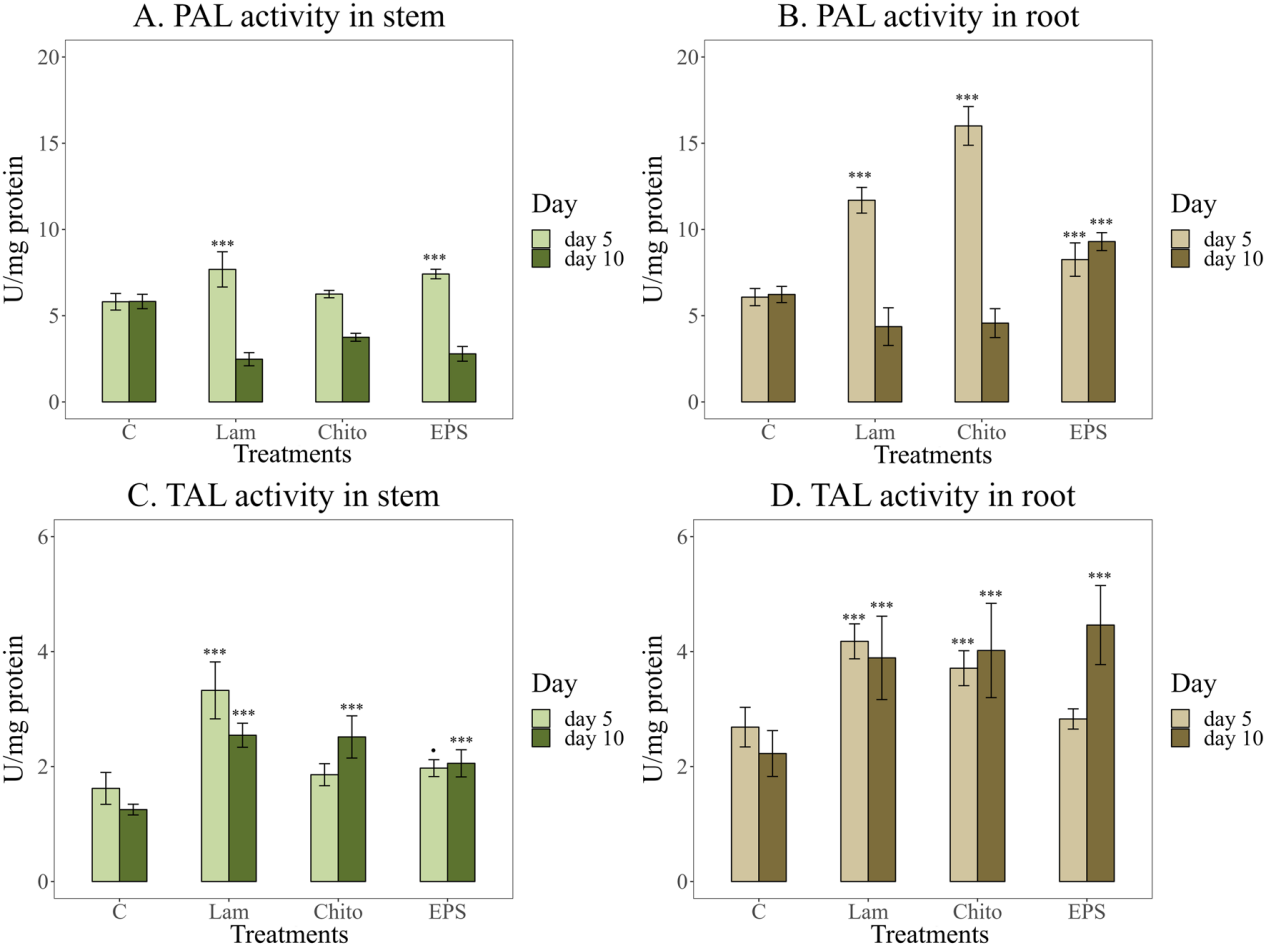
Activity of the phenylpropanoid pathway enzymes after stimulation with the elicitors tested (*C* water control, *Lam* laminarin, *Chito* chitosan, *EPS* EPS from Th32Ag3 strain), **A, B**. Phenylalanine lyase activity (PAL); **C, D**. Tyrosine lyase activity (TAL) after 5 and 10 days of plant growth. Significance against control determined by Dunnett's test, where p value is: *** 0.001; ** 0.01; * 0.05;**.** 0.1. Laminarin and EPS had the strongest stimulatory effect on PAL activity in stems, whereas laminarin and chitosan enhanced PAL activity most strongly in roots. For TAL, laminarin was most effective in stems, while EPS induced the highest activity in roots

EPS significantly increased the activity of marker enzymes of plant resistance pathways. A significant increase in the activity of phenylalanine lyase (PAL) was observed after seed stimulation with elicitors in 5-day-old plants (Fig. 5A). However, this activity was more pronounced in wheat roots. EPS stimulation caused a significant increase in PAL activity in 5-day-old wheat stems (7.4 U/mg protein), similar to the commercial elicitor laminarin (Lam) (7.7 U/mg protein). A several-fold increase in PAL activity was also observed in 10-day-old wheat roots after seed inoculation with EPS compared to the commercial elicitors and water control (Fig. 5B). Tyrosine lyase (TAL) activity was also twofold higher in wheat roots than in stems. EPS increased the activity of this enzyme in wheat stems, similar to the commercial elicitors Lam and chitosan (Chito) (Fig. 5C). In wheat roots, inoculation with EPS resulted in significant TAL activity only in 10-day-old seedlings (Fig. 5D).

**Fig. 6:**
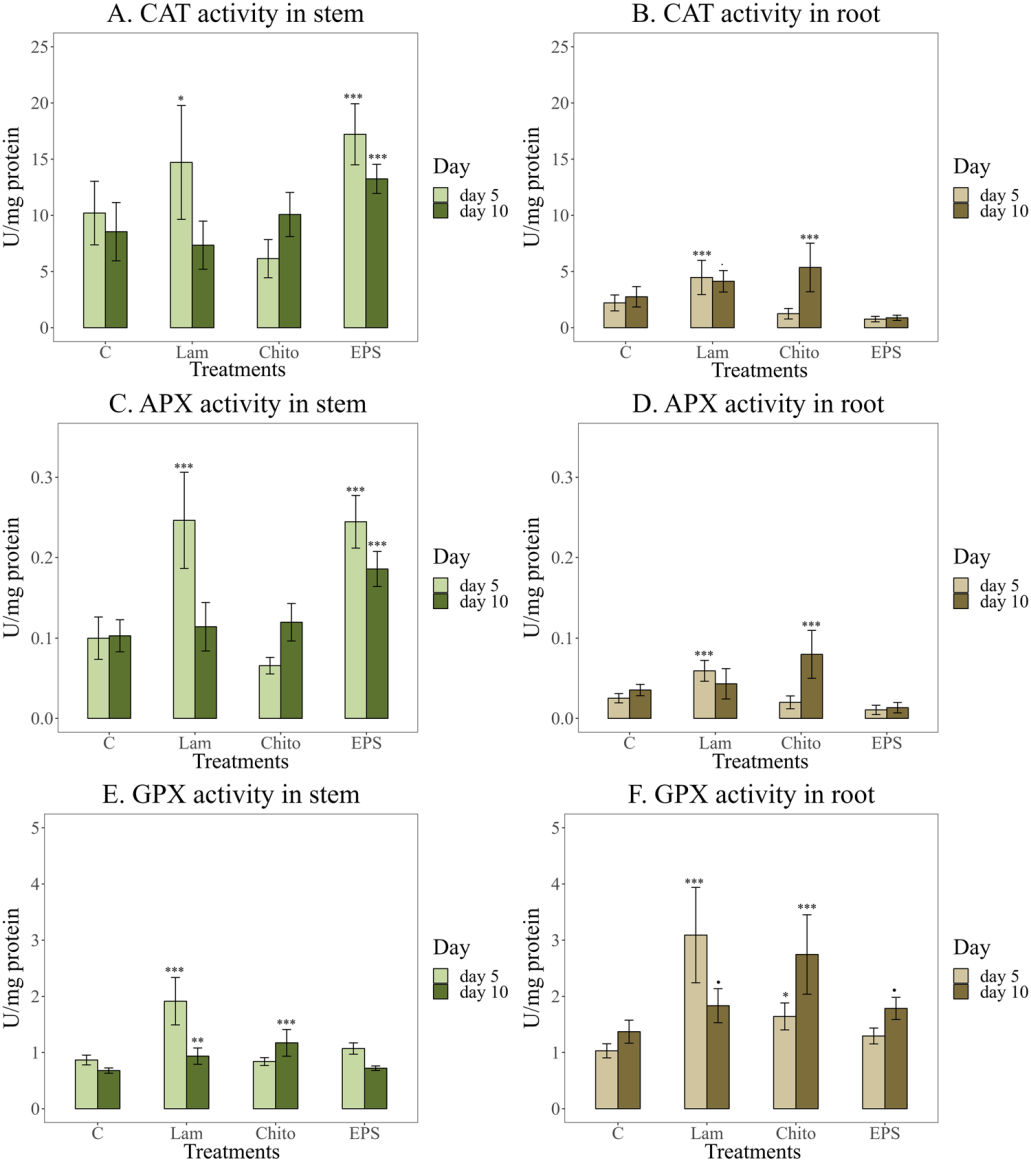
Activity of the antioxidants enzymes after stimulation with the elicitors tested (C—water control, Lam—laminarin, Chito—chitosan, EPS—EPS from Th32Ag3 strain), **A, B**. Catalase (CAT); **C, D**. Ascorbate peroxidase (APX); **E, F**. Guaiacol peroxidase (GPX) after 5 and 10 days of plant growth. Significance against control determined by Dunnett's test, where p value is: *** 0.001; ** 0.01; * 0.05;**.** 0.1. EPS induced the strongest increase in CAT and APX activity in stems, whereas chitosan had the greatest effect in roots. For GPX, laminarin was the most effective elicitor

Both catalase (CAT) and ascorbate peroxidase (APX) activities were much higher in wheat stems (Fig. 6 A, B, C, D). The effect of EPS on wheat stems was similar to that of the commercial elicitor Lam, significantly increasing CAT and APX activity in 5-day-old plants. On the 10th day of growth, the effect of EPS on the increase in the activity of these enzymes was lower than that on the 5th day but was the strongest among all the elicitors used (Fig. 6 A, B, C, D). In turn, the obtained EPS did not significantly affect the increase in guaiacol peroxidase (GPX) activity in wheat stems and roots (Fig. 6 E, F).

**Fig. 7:**
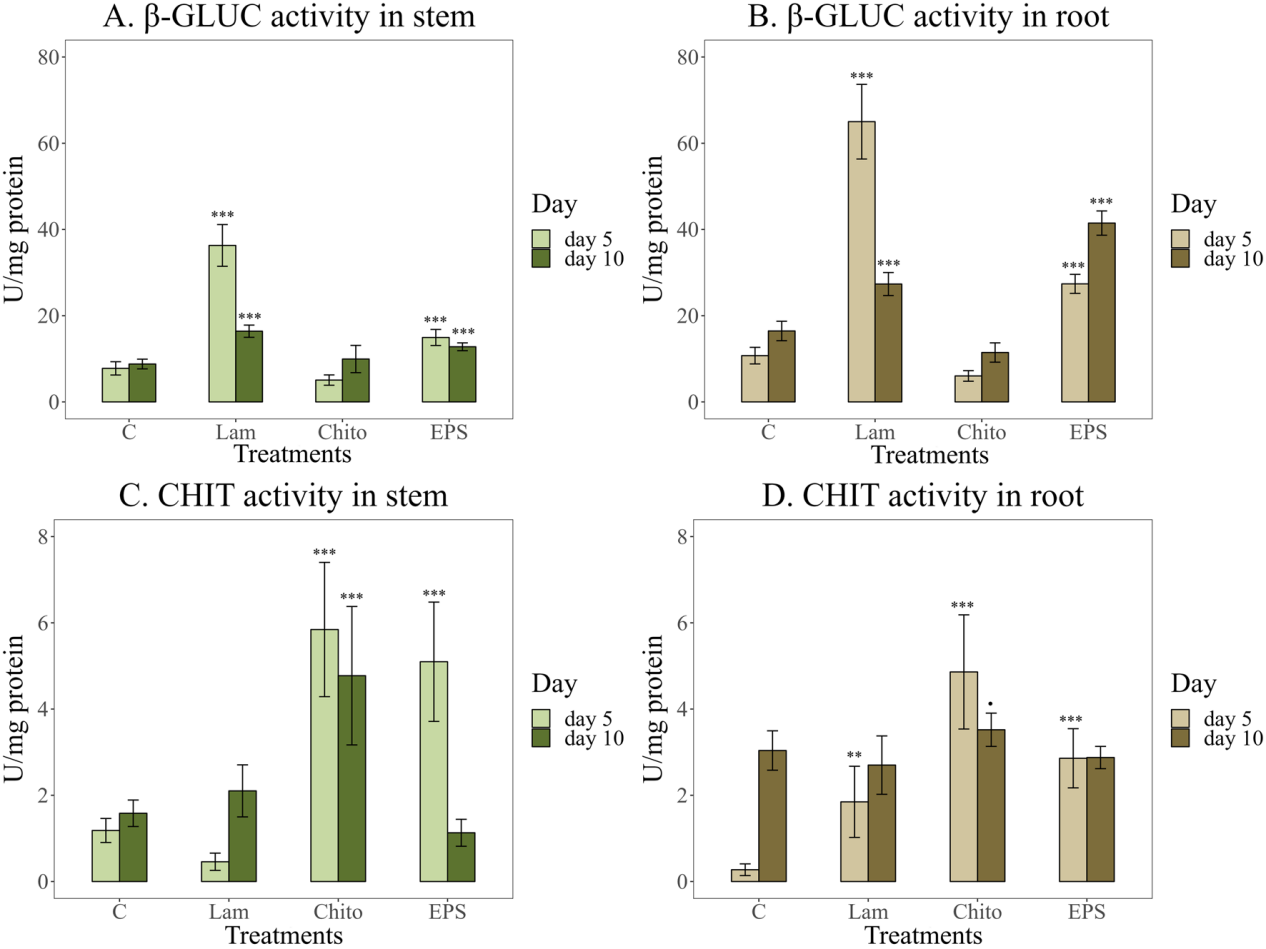
Activity of the pathogenesis related (PR) enzymes after stimulation with the elicitors tested (C—water control, Lam—laminarin, Chito—chitosan, EPS—EPS from Th32Ag3 strain), A,B. β-glucanase(β-GLUC); C,D. Chitinase (CHIT) after 5 and 10 days of plant growth. Significance against control determined by Dunnett's test, where p value is: *** 0.001; ** 0.01; * 0.05;**.** 0.1. β-Glucanase activity was most strongly induced by laminarin in both stems and roots, whereas chitosan caused the greatest increase in chitinase activity

Application of EPS caused a significant increase in β-glucanase (β-GLUC) activity in wheat tissues compared to the water control (Fig. 7A, 7). The increase in β-GLUC activity was significantly higher in wheat roots. Both EPS and Lam caused a several-fold increase in the activity of this enzyme compared to Chito and the water control. However, the obtained EPS had the strongest effect on the increase in β-GLUC activity in 10-day-old roots (41.5 U/mg protein) (Fig. 7B). A significant increase in chitinase (CHIT) activity after EPS application was observed only in 5-day-old stems. This effect was similar to that of the commercial elicitor Chito and almost five times higher than that of the water control (Fig. 7C). After EPS application, CHIT activity in wheat roots was significantly higher than that in the water control after 5 days of culture. However, EPS did not significantly affect the increase in CHIT activity in 10-day-old wheat roots (Fig. 7D).

### Statistical analysis

#### Principal component analysis (PCA) for the optimization parameters of EPS synthesis and plant resistance-inducing properties

**Fig. 8:**
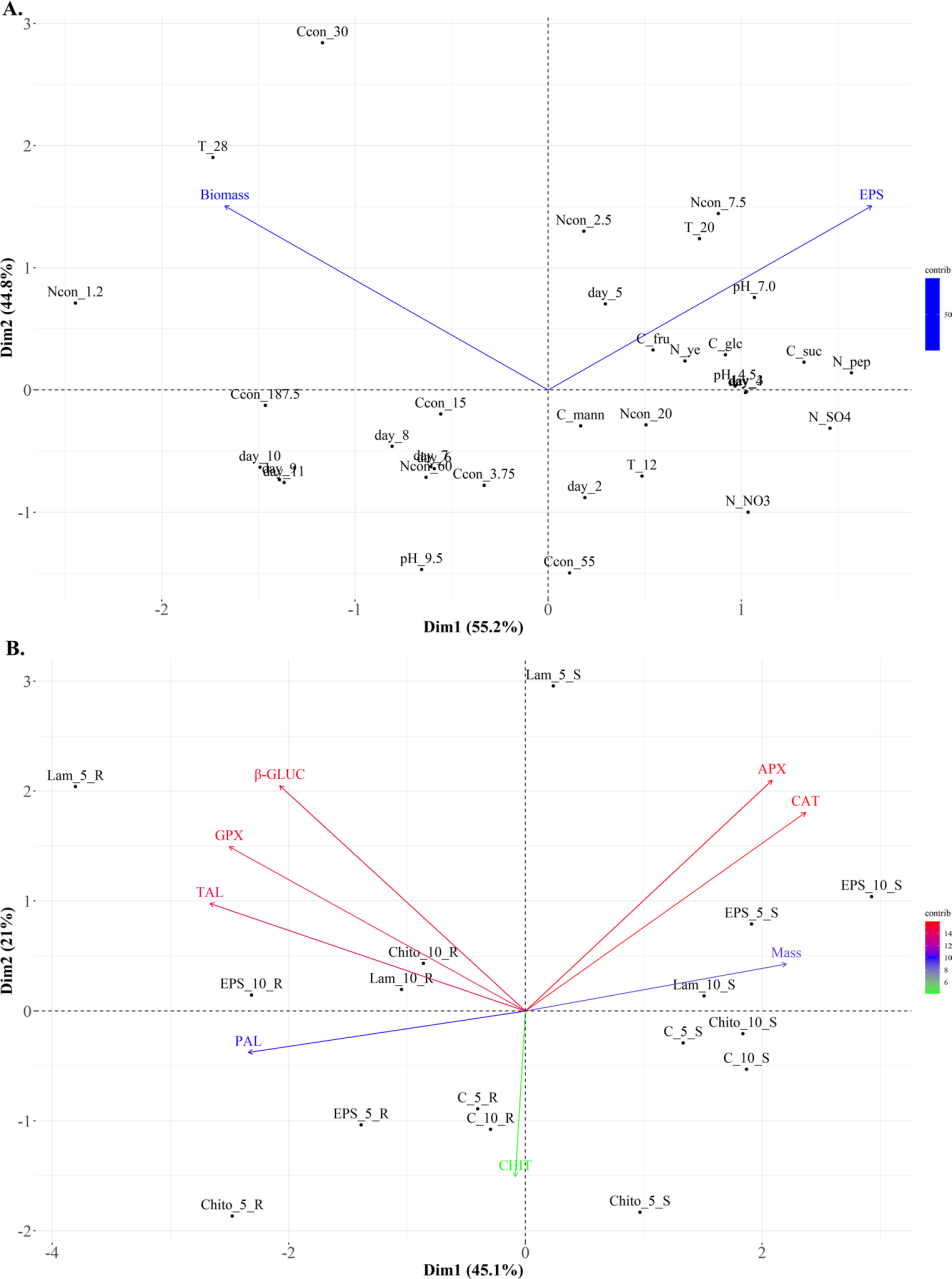
Biplot diagram of Principal Component Analysis (PCA) for: **A** biomass and optimization parameters of EPS synthesis by *S. strictum* strain growing on the medium with different carbon source: sucrose (C_suc), glucose (C_glc), fructose (C_fru), mannose (C_mann) and nitrogen source: peptone (N_pep), yeast extract (N_ye), NH_4_NO_3_ (N_NO_3_), and (NH_4_)_2_SO_4_ (N_SO_4_) with different concentration of carbon source (3.75, 15, 30, 55, 187.5 g/L) and nitrogen source (1.2, 2.5, 7.5, 20, 60 g/L) analyzed in ten days of incubation (2, 3, 4, 5, 6, 7, 8, 9, 10 and 11 days) at three temperatures (12, 20, 28 °C) and three pH value (4.5, 7.0, 9.5); **B** activity of plant resistance enzymes in wheat stems (S) and roots (R) after treatment with obtained EPS, commercial elicitors: laminarin (Lam) and chitosan (Chito), and water control (C) analyzed in two days of incubation (5 and 10 days). *PAL* phenylalanine lyase, *TAL* tyrosine lyase, *APX* ascorbate peroxidase, *GPX* guaiacol peroxidase, *CAT* catalase, *β-GLUC* glucanase, *CHIT* chitinase

The correlation between the amount of EPS and various optimisation parameters was determined by Principal Component Analysis (PCA). The diagram shows the influence of the culture day (day), carbon (C) and nitrogen (N) sources, temperature (T), pH (pH), and concentration of the carbon (Ccon) and nitrogen (Ncon) sources on the biomass and efficiency of EPS synthesis by the *S. strictum* strain (Fig. 8A). The first two dimensions of the PCA explained 100% of the total variation, with principal component 1 (Dim1) accounting for 55.2% and principal component 2 (Dim2) accounting for 44.8% of the variance. A clear cluster of factors influencing the increased synthesis of EPS by the tested strains was noted. Increased EPS synthesis was positively related to the third (day_3) and fourth (day_4) days of incubation. The efficiency of EPS synthesis was influenced by the sources of carbon and nitrogen, with the highest EPS production recorded after using glucose (C_glc), fructose (C_fru), and sucrose (C_suc) as carbon sources, as well as peptone (N_pep) and yeast extract (N_ye) as nitrogen sources. In addition, the nitrogen source concentration had a positive effect on ESP production at concentrations of 2.5 g/L (Ncon_2.5) and 7.5 g/L (Ncon_7.5). Based on the PCA, it can be concluded that the optimal temperature and pH for EPS production by the tested strain were 20 °C (T_20) and 7.0 (pH_7.0), respectively. In turn, the obtained mycelium biomass (biomass) was positively correlated with temperature, reaching the highest amount at 28 °C (T_28). In addition, a nitrogen source with a concentration of 1.2 g/L (Ncon_1.2) and a carbon source with a concentration of 30 g/L (Ccon_30) significantly influenced biomass growth. The remaining parameters did not significantly affect biomass or EPS production by the tested strains. It is also worth emphasising that biomass and EPS were negatively correlated; EPS production was higher in cultures where less biomass was obtained. The negative correlation between biomass and EPS yield suggests that the studied strain allocates carbon and nitrogen resources to either vegetative growth or secondary metabolite synthesis, but not to both processes simultaneously. In our study, increasing the levels of sucrose and peptone led to an improvement in EPS synthesis efficiency without any visible increase in biomass yield.

 The dependence between the obtained EPS and its plant resistance-inducing properties was assessed by Principal Component Analysis (PCA). The diagram shows the effect of EPS and commercial elicitors on the increase in the activity of plant resistance enzymes in wheat stems and roots, depending on the day of cultivation (Fig. 8B). The first two dimensions of the PCA explained 66.1% of the total variation, with principal component 1 (Dim1) accounting for 45.1% and principal component 2 (Dim2) accounting for 21% of the variance. A clear cluster based on the activation of plant resistance markers was observed in different parts of the plant. Activation of PAL, TAL, GPX, and PR proteins (β-GLUC and CHIT) by EPS was higher in wheat roots, similar to commercial elicitors. Moreover, PAL, TAL, GPX, and β-GLUC levels were positively correlated. In turn, APX and CAT activities were higher in wheat stems. EPS also caused an increase in the mass of wheat stems, which correlated positively with APX and CAT. Based on PCA, there was no clear relationship between enzyme activity and the day of culture. However, enzyme activity depends on the tissue. The greatest increase in PAL, TAL, GPX, and β-gluc activity was observed in wheat roots. This suggests that the roots are the primary sites for EPS recognition. This reflects the action of commercial elicitors and confirms that EPS acts as the initial contact elicitor between the tested strain and the plant.

#### Dunnett's test for EPS activity compared to commercial elicitors

The obtained EPS showed a higher capacity to activate plant resistance pathways than the aqueous control, and was more effective than commercial elicitors. The EPS obtained from the culture of the test strain resulted in a statistically (p < 0.05) higher increase in the activity of PAL, CAT, APX, GPX, and β-GLU enzymes than commercially used chitosan. An increase in activity was observed after both 5 and 10 d of plant growth, with the greatest increase observed in wheat stems (Table 5B). Compared to laminarin, statistically significant increases in enzyme activity were observed in fewer cases, mainly after 10 d of plant growth (Table 5A).

**Table 5:** Significance increase of enzymes activity between positive control (laminarin, chitosan) and EPS obtained from strain Th32Ag3 determined by Dunnett's test, where p value is: *** 0.001; ** 0.01; * 0.05;**.** 0.1;—no significance

Enzymatic activity	Positive Control							
	Laminarin				Chitosan			
	Stem		Root		Stem		Root	
	5 days	10 days	5 days	10 days	5 days	10 days	5 days	10 days
PAL	–	–	–	***	***	–	–	***
TAL	–	–	–	–	–	–	–	–
CAT	–	***	–	–	***	***	–	–
APX	–	***	–	–	***	***	–	–
GPX	–	–	–	–	***	***	–	–
β-GLUC	–	–	–	***	***	*	***	***
CHIT	***	–		–	–	–	–	–

## Discussion

The efficiency of EPS synthesis by the *Sarocladium* strictum strain increased by 40%, from 0.83 g/L to 1.17 g/L. The optimum cultivation time for the strain was short, only three days. In contrast, the maximum EPS synthesis capacity of other Ascomycota strains ranged from 8 to 12 days for *Plenodomus* sp., 7 to 10 days for *Penicillium* sp., and approximately 10 days for *Trichoderma* sp. (Huang et al. 2012; Chen et al. 2013; Osińska-Jaroszuk et al. 2015; Nowak et al. 2023). During the cultivation of *S. strictum*, inhibition of EPS synthesis occurred in the first stage after five days of culture growth. This may be influenced by factors ranging from initial growth parameters to changes in strain metabolism over time. An increase in biomass was observed in later days of cultivation, indicating the possible use of EPS as a C- and N-containing reserve substance. In a study on *Ganoderma lucidum*, EPS was obtained every 2 days of cultivation, suggesting cyclical metabolism during cultivation (Asadi et al. 2021). The literature shows that the highest EPS synthesis yields were achieved on substrates with high carbon and nitrogen concentrations, with nitrogen being predominantly organic (Mahapatra and Banerjee 2013; Osińska-Jaroszuk et al. 2015; Barcelos et al. 2020). These conditions were confirmed in our study, where the highest EPS yield in *S. strictum* cultures was obtained on medium containing sucrose (30 g/L) and peptone (7.5 g/L) at pH 7.0 and 20 °C. A medium with a similar composition was optimal for *F. culmorum* strains (Jaroszuk‐Ściseł et al. 2020). The *Trichoderma pseudokoningii* strain showed optimal growth on a medium containing glucose and potato extract (Wang et al. 2016). Some strains require multiple carbon and nitrogen sources to synthesise EPS. The *Penicillium griseofulvum* strain needs three carbon sources (maltose 20 g/L, glucose 10 g/L, and mannitol 20 g/L) and three nitrogen sources (monosodium glutamate 10 g/L, yeast extract 3 g/L, and maize paste 1 g/L), yet shows low EPS synthesis efficiency (0.25 g/L) and a long culture period (30 days) (Chen et al. 2013). Compared to other fungal species, the *S. strictum* Th32Ag3 strain synthesises EPS at levels typical of environmental Ascomycota isolates. The synthesis efficiency of other representatives varies, with EPS obtained from *Plenodomus* sp. cultures at levels of 0.3–0.4 g/L and from *Fusarium clumroum* at levels of 0.2–1.1 g/L (Jaroszuk‐Ściseł et al. 2020; Nowak et al. 2023). Cultures of *Cordyceps militaris* and *Trichoderma koningiopsis* produced a yield of ~ 1 g/L, which is comparable to our results (Lin et al. 2023; Nowak et al. 2025). However, yields exceeding 10 g/L are characteristic of bacteria or modified strains, such as *Aureobasidium pullulans*, where the resulting polymers are used commercially on a large scale (Kumari et al. 2025).

During the optimisation process, it is important to select the appropriate methodology. Modelling using RSM indicated better relationships between cultivation parameters, especially between cultivation conditions (temperature and pH) and medium composition (concentration and source of C and N). However, this requires a greater number of repetitions, which makes up a more complex experiment (Kutyła et al. 2024). In the case of preliminary optimisation in terms of increasing the EPS obtained, it is better to use the one-factor-at-a-time (OFAT) method, which does not indicate the full relationships between factors but shows trends in the culture (Venkatachalam et al. 2021). This is important because scaling up the culture (from a flask to a bioreactor) also results in a decrease in the amount of EPS obtained; therefore, the limited number of optimisation parameters reduces the time and cost of large-scale optimisation (Ferreira Filho et al. 2025). This is particularly important in the context of limited resources and number of bioreactors.

The sugar core constituted the largest proportion of the EPS, representing over 56% of the total composition. GC–MS analysis indicated that the main components were glucose, mannose, and galactose. The notable presence of N-acetylglucosamine was significant, as this monomer forms chitin. It can be hypothesised that the polymers obtained are a mixture of sugar and chitin chains, as evidenced by the effect on chitinase and β-glucanase activities in wheat tissues (Shamshina et al. 2020). The presence of glucosamine residues may improve recognition by plant receptors, which are associated with the stimulation of defense pathways. LysM receptors (CERK1/CEBiP), for example, are crucial for recognising chitin oligosaccharides and initiating defense signalling (Yang et al. 2022). Unlike pure glucans, heterogeneous polymers can modulate immunity at various levels (Yun et al. 2023). The presence of uronic and amino acids significantly affected the physicochemical properties of the resulting polymers. Uronic acids, for instance, affect the charge of the molecule, thereby altering its solubility, facilitating its penetration through the cell wall, and improving its contact with receptors (Wang et al. 2023). The structure and properties of the polymers described are influenced by various factors, including culture conditions. Fraga et al. (2014) showed that varying culture parameters (initial pH, source of C and N) influenced the quantitative content of fucose, galactose, glucose and mannose in the EPS synthesised by *G. lucidum*. The changes were quantitative but not qualitative. The EPS contained all four sugar monomers, with pH value and peptone concentration being the most significant influences on EPS composition. Silva et al. (2020) observed a similar relationship in EPS from *Arthrospira platensis* cultures, where NaNO_3_ content and light affected the ratio between carbohydrates and proteins. This finding confirms that culture parameters influence quantitative alterations in the EPS. The soluble fraction of the EPS included sugars as the dominant part, along with proteins, uronic acids, and amino sugars. EPSs from *Fusarium* sp., *Cordyceps* sp., and *Beauveria* sp. exhibited a comparable structure (Madla et al. 2005; Jaroszuk‐Ściseł et al. 2020). The resolubility of EPSs after alcohol precipitation has been a challenge in many studies. Madla et al. (2005) described variable solubility of EPS from 16 fungal isolates. Some polymers were fully soluble, whereas others were only partially soluble in water or dimethyl sulfoxide (DMSO). These studies showed that polymer solubility is not dependent on the molecular weight. The EPS obtained in our study consisted of 56% sugar cores linked to uronic acids and fragments composed of N-acetylglucosamine. The sugar core was found to consist of 36% glucose, 15.8% mannose, and 36.2% galactose, classifying it as galacto-glucan. While these monomers are found in most EPS, one of them is often more dominant. For example, the EPS obtained from *C. militaris* cultures consists of a sugar core comprising ~ 56% of the total, with a higher proportion of glucose (56.1%), as well as traces of mannose (24.9%), galactose (19%), and rhamnose (8.9%) (Lin et al. 2023). In contrast, the composition of polymers obtained from *F. culmorum* cultures varied depending on the strain tested and the degree of purification. EPS obtained from PGPF strain cultures after purification consisted of 35.8% glucose, 35% mannose, and 21.9% galactose. For DRMO, the respective percentages were 53.8%, 41.8%, and 4.3%, and for phytopathogens, they were 19.1%, 72.6%, and 19.1% (Jaroszuk‐Ściseł et al. 2020). Many commercial EPSs, on the other hand, are homopolysaccharides consisting mainly of a single subunit, such as glucose in the case of pullulan synthesised by *A. pullulans* (Kumari et al. 2025)*.*

EPS possesses elicitor properties, increasing the activity of plant resistance enzymes. Using laminarin and chitosan as positive controls, the EPS from *S. strictum* showed two-fold higher elicitor activity than chitosan compared to non-treated plants. Eliciting properties of polymeric substances relate to their structure, primarily the degree of polymerisation (DP), with chains above a DP of five showing elicitor properties. Immune stimulation begins when receptors recognise elicitors as pathogen-associated molecular patterns (PAMPs), activating signal transduction through mitogen-activated protein kinases (MAPKs), and leading to increased enzyme activity in plant immunity pathways (Zheng et al. 2020). In the present study, it was demonstrated that the activity of β-glucanase and chitinase increased after stimulation with the obtained polymers in plant tissue. This may indicate direct involvement in the formation of oligomers that stimulate specific pathways in plant tissues. The application of EPS was observed to significantly activate phenylpropanoid pathway enzymes (PAL and TAL), particularly in wheat roots, as evidenced in our study. These enzymes are involved in lignification of the cell wall, which impedes the infestation of plant tissues by phytopathogens (Dong and Lin 2021). The activity of this group of enzymes is affected by the oligosaccharides, glucans, and polymers obtained during algal growth (Paris et al. 2019). A comparable increase in PAL activity was documented following the stimulation of tomatoes with ulvan and oligoulvans, namely polysaccharides derived from the culture of the alga *Ulva Lactuca* (El Modafar et al. 2012). Furthermore, the elicitation of olive leaves with polymers results in an increase in PAL and TAL activities (Aitouguinane et al. 2022). Klarzynski et al. (2000) demonstrated that linear β-glucans also exert beneficial effects on PAL activity in tobacco tissues. The interaction between different fractions of polymers obtained from *Fusarium oxysporum* strain cultures resulted in an increase in PAL activity and flavonoid content in *Fagopyrum tataricum* (L.) (Zhong et al. 2016). Antioxidant enzymes, including CAT, APX, and GPX, are involved in the initial stages of phytopathogen attack and tissue damage and are responsible for regulating reactive oxygen species (ROS) levels(Li et al. 2022). In our study, the activities of antioxidant enzymes (CAT, APX, and GPX) remained unchanged in the roots. However, an increase was observed in the stems. This increase in CAT and APX activities was correlated with increased biomass growth in the stems. This suggests that EPS stimulation may promote local ROS modulation and changes in the balance between the aboveground and belowground parts of the plant in response to stress factors. The action of ROS mechanisms emphasises that CAT, APX, and GPX are independently regulated and respond differently to local H₂O₂ fluxes, as well as to the presence of receptors and secondary signals (Zandi and Schnug 2022). The dose-time response correlation is also important in the context of all described enzymes. In the present study, we observed changes in the enzyme levels over time between days 5 and 10 of wheat growth. These changes also translated into altered enzyme levels in the roots and stems. For example, there was a decrease in β-glucosidase activity in the stems on day 10, whereas chitinase activity increased in the roots at the same time. Literature reports indicate an important relationship between elicitor dose and the time of its action. This translates to the kinetics of the enzyme activity. The effects of elicitors have been shown to depend strongly on both the concentration and time of measurement; for example, laminarin and chitosan exhibit different kinetics of immune gene induction and enzymatic activity (Meresa et al. 2024). Glucans extracted from the *Trichoderma hamatum* strain UOM 13 cell wall have been shown to augment antioxidant enzyme activity in *Pennisetum glaucum* tissues, similar to the effects observed with laminarin and chitosan (Lavanya et al. 2022). Zhong et. al (2016), observed increased antioxidant activity in Tartary buckwheat tissues using extracellular polymers *and F. oxysporum* wall polymer fractions to stimulate immunity. Enzymes with β-glucanase and chitinase activities are categorised as pathogenesis-related (PR) proteins, which are involved in the response to fungal cell wall fragments. The presence of EPS increased these enzyme activities. A similar relationship was found between *T. hamatum* cell wall glucans and β-glucans from *Septoria tritici* cultures (Van Loon et al. 2006; Lavanya et al. 2022). Stimulation of wheat tissues with these elicitors increased marker enzyme activity and, in several cases, increased stem and root fresh weights. A comparable relationship was identified after the administration of glucooligosaccharides (GOS) from *Laetiporus sulphureus* α-glucans and chitooligosaccharides (COS) from chitosan (Li et al. 2022; Nowak et al. 2022). Additional sugar compounds during germination and growth may accelerate respiration and increase photosynthetic pigments, thereby increasing root water uptake and fresh plant weight. Elevated PAL and GPX enzyme activity leads to accelerated formation of cell wall lignification precursors, which may influence fresh weight (Randhir and Shetty 2003). In the short term (5 days), roots exhibited responses such as increased PAL/TAL and activation of β-glucanase/chitinase, indicating a swift recognition of the elicitor where it directly contacts the substrate and the start of local signalling pathways. In contrast, later changes in shoots, such as heightened APX and CAT activity on day 10 and a correlation with stem weight increase, imply the transmission of systemic signals and the activation of ROS control mechanisms at later response stages. This temporal shift—initial strong induction of phenylpropanoid pathways in roots followed by modulation of the antioxidant system in the shoots—aligns with findings from other researchers studying polysaccharide elicitors in wheat and cereals, who noted varying reaction dynamics between tissues and at different times.

These findings indicate that research on the interaction between endophytic fungi and plants offers significant potential for further exploration. The factors influencing these interactions are extensive and extend beyond the scope of the established mechanisms. Therefore, it is imperative to investigate these relationships across diverse disciplines. Obtaining EPS and verifying their elicitor properties serves as a fundamental step in elucidating the precise mechanisms underpinning these interactions., it is imperative to undertake comprehensive studies in diverse fields to elucidate these relationships.

## Conclusions

The EPS obtained from the *S. strictum* Th32Ag3 strain exhibited clear elicitor properties in wheat seedlings, as evidenced by enhanced defense-related enzyme activity (PAL, TAL, β-glucanase, and chitinase). However, the activities of the enzymes from the catalase and peroxidase groups remained unchanged. These results suggest that EPS derived from S. strictum cultures may protect plants against biotic stress. This is important when fungal polymers are used to protect phytopathogenic strains. Furthermore, an increase in the activity of these enzymes was observed at concentrations as low as 0.05%. Polymers that require low concentrations to induce plant resistance can be used to protect wheat against phytopathogens in phytotrons and field studies. Another significant outcome of the research was the enhancement of EPS synthesis efficiency to 1.17 g/L.

## Supplementary Information

Below is the link to the electronic supplementary material.

Supplementary file1 (DOCX 246 KB)

## Data Availability

Data will be made available on request.
