# Supplementary material for: Characterisation and elicitor properties of extracellular polymeric substances (EPS) obtained from cultures of Sarocladium strictum isolated from rye rhizosphere
Source: Arch Microbiol. 2025 Oct 21;207(12):323. doi: 10.1007/s00203-025-04520-y (PMC12540626; doi:10.1007/s00203-025-04520-y)
Supplement: Supplementary file 1 — Supplementary file1 (DOCX 246 KB) [file 203_2025_4520_MOESM1_ESM.docx]

**Title**

Characterisation and elicitor properties of extracellular polymeric substances (EPS) obtained from cultures of *Sarocladium strictum* Th32Ag3

**Authors and affiliations**

Artur Nowak^1*^, Renata Tyśkiewicz^2^, Iwona Komaniecka^3^, Anna Pawlik^4^, Grzegorz Janusz^4^, Jolanta Jaroszuk-Ściseł^1^

^1^Department of Industrial and Environmental Microbiology, Institute of Biological Sciences, Maria Curie-Skłodowska University, Akademicka 19, 20-033 Lublin, Poland; artur.nowak@mail.umcs.pl; [jolanta.jaroszuk-scisel@mail.umcs.pl](mailto:jolanta.jaroszuk-scisel@mail.umcs.pl)

^2^Analytical Laboratory, Łukasiewicz Research Network – New Chemical Syntheses Institute, Tysiąclecia Państwa Polskiego Ave. 13A, 24-110, Puławy, Poland, renata.tyskiewicz@ins.lukasiewicz.gov.pl

^3^Department of Biochemistry and Biotechnology, Institute of Biological Sciences, Maria Curie-Skłodowska University, Akademicka 19, 20-033 Lublin, Poland; grzegorz.janusz2@mail.umcs.pl, anna.pawlik@mail.umcs.pl

^4^Department of Genetics and Microbiology, Institute of Biological Sciences, Maria Curie-Skłodowska University, Akademicka 19, 20-033 Lublin, Poland; iwona.komaniecka@mail.umcs.pl

*** Corresponding author**

^*^Artur Nowak

^1^Department of Industrial and Environmental Microbiology, Institute of Biological Sciences, Maria Curie-Skłodowska University

Akademicka 19, 20-033 Lublin, Poland

artur.nowak@mail.umcs.pl


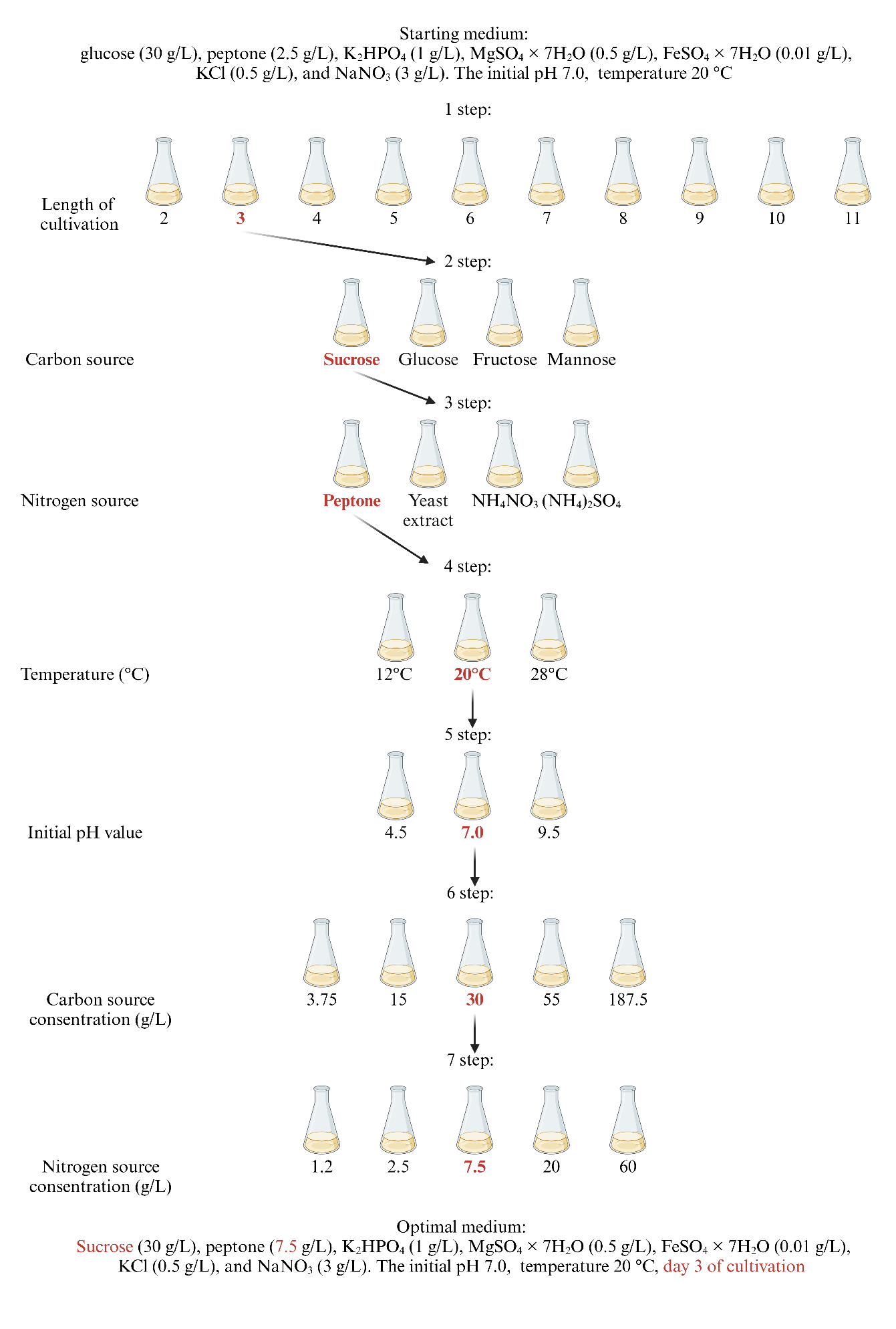


Fig. S1. Step-by-step optimisation diagram showing the different steps and the optimal breeding parameters obtained (red). Each optimal parameter obtained in the previous steps was used in the next steps. Created in BioRender.com.

Table S1. Results of step-by-step optimisation of S. strictum strain culture on the biomass obtained (g/L). Statistical analysis was performed by Anova test using Tukey post hoc p<0.05.

|  | **Optimised parameter** | | | | | | | | | | | | | | | | | |
| --- | --- | --- | --- | --- | --- | --- | --- | --- | --- | --- | --- | --- | --- | --- | --- | --- | --- | --- |
| **Biomass**  **g/L** | **Day of culture** | | | | | | | | | | | | | | | | | |
|  | 2 | 3 | 4 | | | 5 | | 6 | | 7 | 8 | | | 9 | | 10 | | 11 |
|  | 8.82±0.05  c | 8.91±0.46  bc | 8.91±0.32  bc | | | 13.17±0.22  a | | 11.81±0.47  abc | | 11.91±2.77  abc | 12.98±0.07  a | | | 13.9±0.17  a | | 14.49±0.96  a | | 13.76±1.27  a |
|  | **Carbon source** | | | | | | | | | | | | | | | | | |
|  | Sucrose | | | | Glucose | | | | | Fructose | | | | | Mannose | | | |
|  | 8.75±0.25  c | | | | 10.12±0.64  b | | | | | 11.33±0.03  a | | | | | 11.61±0.42  ab | | | |
|  | **Nitrogen source** | | | | | | | | | | | | | | | | | |
|  | Peptone | | | | Yeast extract | | | | | NH_4_NO_3_ | | | | | (NH_4_)_2_SO_4_ | | | |
|  | 7.77±0.03  b | | | | 10.59±0.56  a | | | | | 5.99±0.31  c | | | | | 6.76±0.25  c | | | |
|  | **Temperature (°C)** | | | | | | | | | | | | | | | | | |
|  | 12 | | | | | | 20 | | | | | | 28 | | | | | |
|  | 8.47±0.28  c | | | | | | 13.3±0.49  b | | | | | | 22.65±0.55  a | | | | | |
|  | **Initial pH value** | | | | | | | | | | | | | | | | | |
|  | 4.5 | | | | | | 7.0 | | | | | | 9.5 | | | | | |
|  | 9.22±0.04  b | | | | | | 11.05±0.47  a | | | | | | 9.58±0.72  b | | | | | |
|  | **Carbon source concentrations (g/L)** | | | | | | | | | | | | | | | | | |
|  | 3.75 | | | 15 | | | | | 30 | | | 55 | | | | | 187.5 | |
|  | 10.56±0.49  d | | | 13.02±0.92  c | | | | | 23.74±0.94  a | | | 7.25±0.58  e | | | | | 15.89±0.42  b | |
|  | **Nitrogen source concentrations (g/L)** | | | | | | | | | | | | | | | | | |
|  | 1.2 | | | 2.5 | | | | | 7.5 | | | 20 | | | | | 60 | |
|  | 21.24±0.96  a | | | 15.25±0.96  b | | | | | 13.62±0.7  bc | | | 9.64±0.64  d | | | | | 11.73±0.31  c | |
